# Model-informed drug development in public health emergency of international concern: accelerating marketing authorization of simnotrelvir

**DOI:** 10.1128/aac.00614-25

**Published:** 2025-09-18

**Authors:** Bu-Fan Yao, Yang Yang, Shan-Sen Xu, Bo-Hao Tang, Jia Chen, Zi-Jia Guo, Hong-Lin Hu, Wei Zhang, Shu-Meng Fu, Xin-Fang Zhang, Guo-Xiang Hao, Xin-Mei Yang, Lin-Lin Song, Pan-Pan Ye, Lian Liu, Shun-Wei Zhu, Yi Zheng, Wei Zhao

**Affiliations:** 1 Department of Clinical Pharmacy, Key Laboratory of Chemical Biology (Ministry of Education), School of Pharmaceutical Sciences, Cheeloo College of Medicine, Shandong University 600582 , Jinan, China; 2 State Key Laboratory of Neurology and Oncology Drug Development 675171 , Nanjing, China; 3 Simcere Zaiming Pharmaceutical Co., Ltd. 671598 , Nanjing, China; 4 Jiangsu Simcere Pharmaceutical Co., Ltd, Nanjing, China; 5 Department of Clinical Pharmacy, Shandong Engineering and Technology Research Center for Pediatric Drug Development, Shandong Medicine and Health Key Laboratory of Clinical Pharmacy, The First Affiliated Hospital of Shandong First Medical University & Shandong Provincial Qianfoshan Hospital 66310, Jinan, China; IrsiCaixa Institut de Recerca de la Sida, Barcelona, Spain

**Keywords:** public health emergency, model-informed drug development, simnotrelvir, nonlinear mixed-effects model, exposure-response analysis, Omicron variant

## Abstract

During a Public Health Emergency of International Concern (PHEIC), rapid drug development is critical, but traditional clinical trials are time-consuming and high-risk. This study used coronavirus disease 2019 (COVID-19) as an example to highlight the pivotal role of model-informed drug development (MIDD) in expediting the marketing authorization of simnotrelvir in China, a 3CL^pro^ inhibitor for COVID-19 treatment. Three simnotrelvir clinical trials (Phase Ia, Ib, II/III) were optimized using the MIDD approach. Pharmacokinetics was investigated using nonlinear mixed-effects models, exposure was calculated through Monte Carlo simulation and Bayes estimation, and exposure-efficacy/safety relationships were analyzed using linear/logistic regression models. The MIDD approach began with a translational study to predict patients’ starting doses using first-in-human data, preclinical data, and pharmacodynamic surrogate marker. A dose level of 750 mg/100 mg simnotrelvir/ritonavir was recommended, using simulation results with 90.6% of participants’ trough concentration exceeding EC_90_ for anti-Omicron variant. Then, a biomarker confirmation study to investigate dose-exposure-response relationship found that 750 mg suppressed viral load more than 300 mg (−4.995 vs. −4.236 log_10_ copies/mL, *P* = 0.0367) in Phase Ib. Finally, a randomized controlled trial to confirm benefit-risk ratio found that simnotrelvir/ritonavir reduced time to sustained resolution of 11 clinical syndromes by 1.5 days compared with placebo, had no serious adverse events, and had a flat exposure-response relationship with viral load reduction, time to sustained resolution, and ≥2 grade treatment-emergent adverse event rate with approved dosage. MIDD enhanced clinical trial success, optimized the benefit-risk profile, and expedited marketing authorization for new drug development in response to PHEIC.

**CLINICAL TRIALS:**

This study is registered with ClinicalTrials.gov as NCT05339646, NCT05369676, and NCT05506176.

## INTRODUCTION

A Public Health Emergency of International Concern (PHEIC) poses a serious threat to global human health, often characterized by rapid transmission, high morbidity, and significant mortality. The development of novel therapeutics targeting the causative pathogens is a crucial strategy to curb the spread of such outbreaks. The COVID-19 pandemic, for instance, has placed an immense burden on global public health. As of April 2025, WHO data reported 777 million confirmed cases and 7.09 million deaths worldwide (1). The rapid spread of coronavirus disease 2019 (COVID-19), driven by emerging variants, such as Omicron and its evolving subtypes, underscores the ongoing challenges in controlling the virus. However, traditional clinical trial approaches of drug development are often time-consuming and have a high risk of failure, making them unsuitable for the urgent development of effective treatments. This study uses COVID-19 as a case example to explore strategies for accelerating the regulatory approval of novel therapeutics during a PHEIC.

Scientists from industry, academia, and regulatory agencies have collaborated to expedite the creation of safe and effective anti-COVID-19 drugs. Model-informed drug development (MIDD) uses quantitative models to inform clinical trial design and accelerate drug development. The number of MIDD applications over time has steadily increased in recent years, and MIDD approaches have been applied across multiple therapeutic areas, including infectious diseases (2–4). It integrates findings from preclinical and early clinical trials to address critical issues in drug development, making it particularly useful for guiding therapeutic interventions against COVID-19.

Simnotrelvir (also known as SSD8432/SIM0417), a 3-chymotrypsin-like protease (3CL^pro^) inhibitor, co-packaged with ritonavir, received conditional approval from the National Medical Products Administration (NMPA) in China in January 2023 for treatment of COVID-19. In July 2024, simnotrelvir/ritonavir became the first anti-COVID-19 drug in China to receive regular approval. The entire clinical development process of simnotrelvir, from first-in-human trials to marketing authorization, was completed within 9.8 months, which was significantly faster than typical clinical trial timeline (5). This accelerated development was facilitated by adaptive trial designs supported by MIDD, which enabled the accurate recommendation of safe and effective dose regimens for clinical trials. The choice of the MIDD approach depends on the specific objectives of the program, and population pharmacokinetic (PopPK) model is a valuable tool (6–8). Therefore, this study aimed to highlight the critical role of the MIDD approach in accelerating the marketing authorization of simnotrelvir, especially in dose selection for patients infected with the Omicron variant through a series of clinical trials.

## RESULTS

### Transnational study to predict patients’ starting dose

#### Data from first-in-human clinical trial

A total of 60 volunteers from first-in-human trial (Phase Ia) in China were included in PopPK analysis. The median (range) weight and age were 64.4 (46.1–82.9) kg and 30 (20–50) years, respectively (Table 1).

**TABLE 1:** Demographic information of participants included in the model 1 and model 2 PopPK analyses^*a*^

	Model 1	Model 2			
	Phase Ia	Phase Ia^*b*^	Phase Ib	Phase II/III	Total
N (%)	72 (100)	60 (33.0)	24 (19.7)	98 (80.3)	182 (100)
Subjects	Healthy volunteers	Healthy volunteers	Patients	Patients	Healthy volunteers and Patients
Body weight, kg	64.4 (46.1–82.9)	64.9 (46.1–82.9)	68.0 (50.0–100)	63.5 (39.0–115)	65.0 (39.0–115)
Body mass index, kg/m^2^	22.7 (19.4–26.4)	22.8 (19.7–26.4)	24.2 (17.3–29.2)	23.3 (16.2–32.5)	23.2 (16.2–32.5)
Age, years	30 (20–50)	30.5 (20–50)	36.5 (19–67)	39 (18–66)	35 (18–67)
Gender					
Number of female (%)	19 (26.4)	17 (28.3)	4 (16.7)	44 (44.9)	65.0 (35.7)
Creatinine clearance rate, mL/min	117 (72.9–178)	114 (72.9–178)	117 (64.8–177)	113 (71.2–230)	113 (64.8–230)
Lactate dehydrogenase, U/L	145 (111–191)	146 (111–191)	155 (116–211)	188 (117–284)	169 (111–284)
Alkaline phosphatase, U/L	65.5 (24.0–111)	65.5 (24.0–111)	71.2 (45.4–111)	74.5 (41.0–139)	71.0 (24.0–139)
Food status					
Fasted status, N (%)	72 (100)	60 (100)	24 (100)	98 (100)	170 (93.4)
Fed status, N (%)	24 (33.3)	24 (40)	0 (0)	0 (0)	12 (6.59)
Dose regimen					
Single dose, N (%)	42 (58.3)	42 (70.0)	0 (0)	0 (0)	42 (23.1)
Multiple doses, N (%)	30 (41.7)	18 (30.0)	24 (100)	98 (100)	140 (76.9)
Simnotrelvir monotherapy, N (%)	24 (33.3)	0 (0)	0 (0)	0 (0)	0 (0)
Simnotrelvir/ritonavir, N (%)	60 (83.3)	60 (100)	24 (100)	98 (100)	182 (100)

^*a*^ The values are expressed as median (range). N, number of subjects.

^*b*^ The subjects administrated with simnotrelvir/ritonavir combination therapy in Phase Ia clinical trial.

#### Initial model building

**Fig 1:**
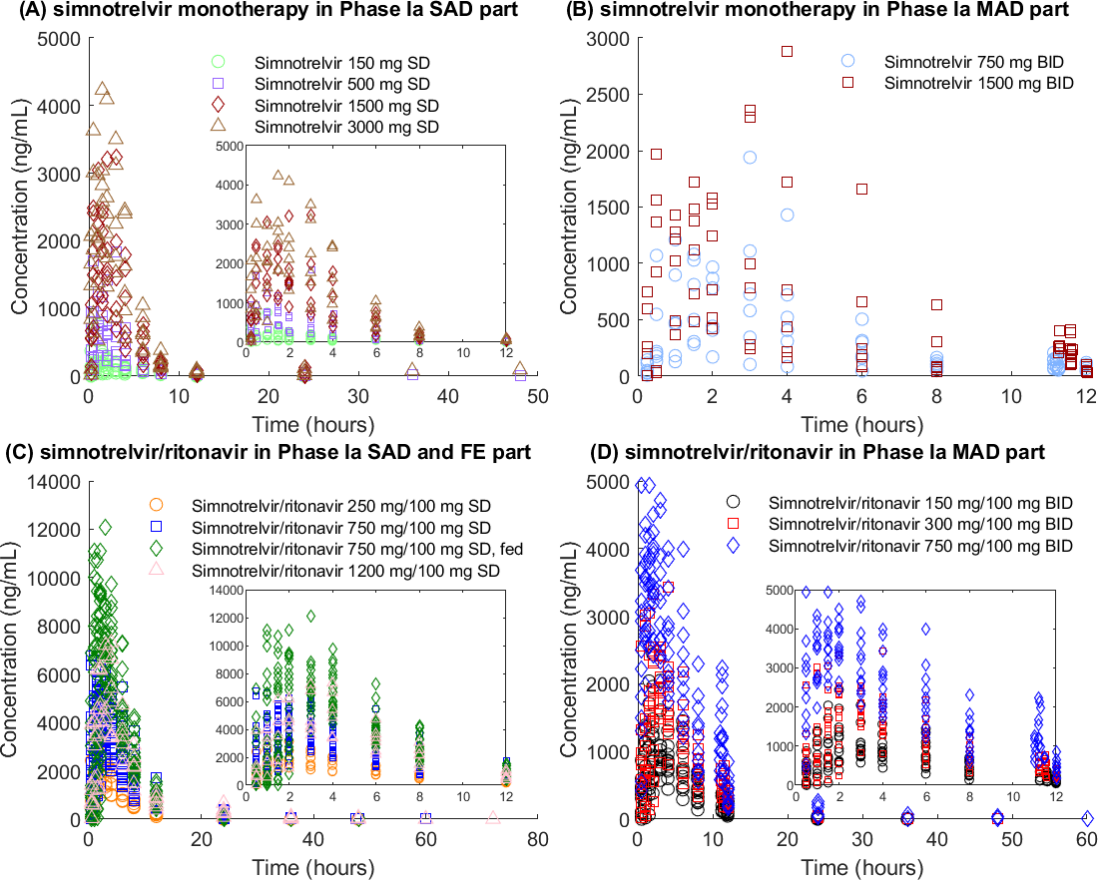
The concentration versus time curve of simnotrelvir from first-in-human trial used in model 1. (**A**) Simnotrelvir monotherapy in Phase Ia single-ascending dose part; (**B**) simnotrelvir monotherapy in Phase Ia multiple-ascending dose part; (**C**) simnotrelvir/ritonavir in Phase Ia single-ascending dose part and food effect part; (**D**) simnotrelvir/ritonavir in Phase Ia multiple-ascending dose part.

**Fig 2:**
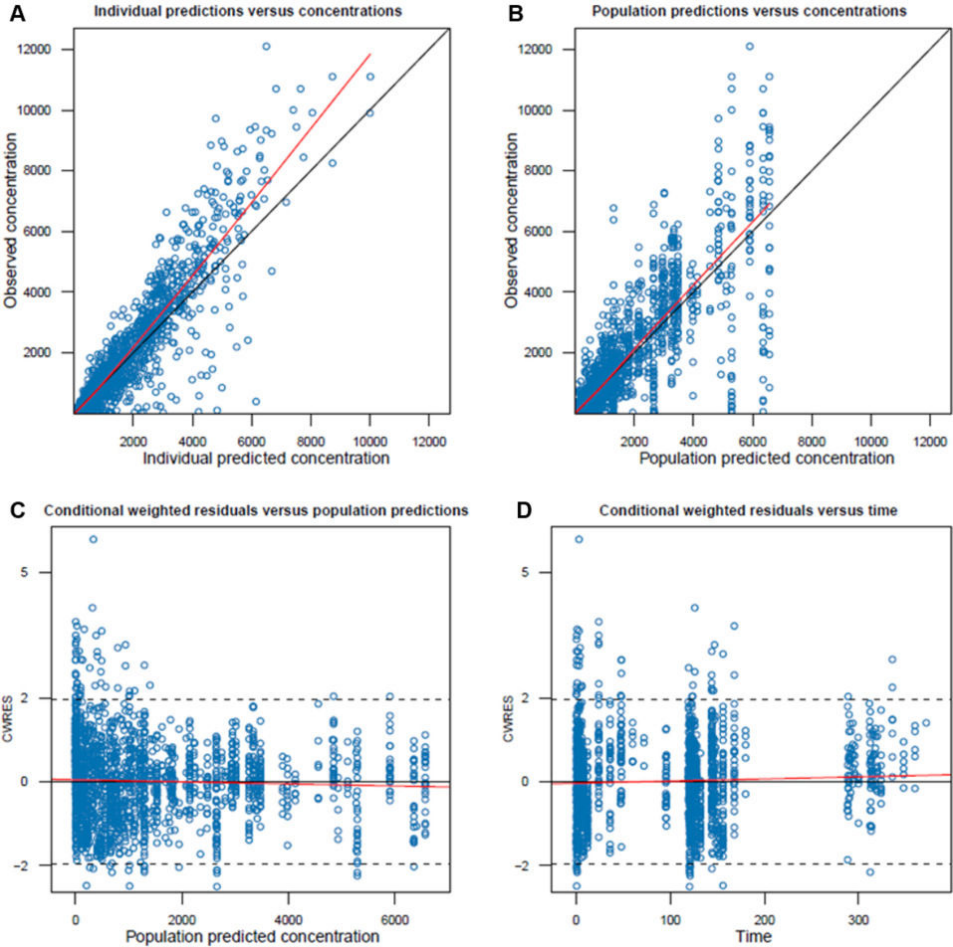
Goodness of fit plots of final simnotrelvir model 1. (**A**) Observed concentration versus individual predicted concentration; (**B**) observed concentration versus population predicted concentration; (**C**) conditional weighted residuals versus population predicted concentration; (**D**) conditional weighted residuals versus time. The red line represented a locally estimated scatterplot smoothing (LOESS) curve, which helped to identify systematic trends or biases between observed data and model predictions. In Fig. 2C and D, the dashed horizontal lines at CWRES = ±2 represented the ±2 standard deviation boundaries. The fact that the vast majority of CWRES values (over 95%) fall within this range supported the assumption of normally distributed residuals and indicated that the model adequately characterized random variability.

A total of 1,742 concentrations (Fig. 1 ) from 60 volunteers were available to build the PopPK model. Detailed model 1 results are presented in the Results in the Supplement and are summarized as follows:

A two-compartment model with first-order absorption best described the data (Fig. 2; Fig. S1 and S2 in the Supplement). In the covariate analysis (Table S2 in the Supplement), the combined use of ritonavir showed a significant effect on CL and F1 compared with simnotrelvir monotherapy. Simnotrelvir dose amount and food status could influence absorption parameters. The median (range) of estimated CL and Ka (Table 2) was 39.05 (26.27–62.16) L/h and 0.20 (0.11–0.29) h^−1^, respectively, when simnotrelvir was co-administrated with ritonavir.

**TABLE 2:** Population pharmacokinetic parameters of simnotrelvir and bootstrap results of model 1 after first-in-human trial^*a*^

Parameters	Final model		Bootstrap (*n* = 1000)		
	Estimate	RSE%	Median	5th	95th
CL (L/h)^*b*^	174	7.0	175	156	198
Ritonavir-CL	0.241	20.6	0.247	0.151	0.342
V2 (L)	33.7	26.3	32.9	18.3	51.2
V3 (L)	3.84	17.5	4.72	0.881	11.2
Q (L/h)	58.6	9.9	76.1	22.3	265
Ka (h^−1^)^*c*^	0.425	22.5	0.435	0.291	0.797
DOSE-Ka^*d*^	−0.127	25.6	−0.130	−0.202	−0.0756
DOSE-Ka^*e*^	−0.138	26.4	−0.142	−0.241	−0.0756
FOOD-Ka	1.49	3.7	1.49	1.40	1.58
F1^*f*^	1	0	1	1	1
FOOD-F1	1.39	3.0	1.39	1.33	1.47
Ritonavir-F1^*g*^	1.36	24.0	1.37	0.852	2.05
Ritonavir-F1^*h*^	1.61	23.2	1.65	1.04	2.41
ALAG (h)	0.223	2.0	0.225	0.215	0.236
Inter-individual variability (CV%)					
CL	27.1	10.6	26.8	21.7	31.3
V2	83.0	21.0	82.0	36.8	115
Ka	19.4	9.7	19.3	16.1	22.8
Inter-occasion variability (CV%)					
V2	69.1	18.8	71.7	47.1	111.2
Residual variability					
Proportional (%)	46.0	3.7	45.6	42.9	48.5
Additive (ng/mL)	1.556	11.1	1.53	1.30	3.33

^*a*^ CL, clearance; V2, central volume of distribution; V3, peripheral volume of distribution; Ka, absorption rate constant; Q, intercompartment clearance; F1, relative bioavailability; ALAG, absorption lag time; DOSE-Ka, effect of dose amount (mg) to Ka; FOOD-Ka, effect of food on Ka (fed vs. fasted); FOOD-F1, effect of food on F1 (fed vs. fasted); Ritonavir-F1, effect of ritonavir administration time on F1 (prior vs. simultaneous with simnotrelvir).

^*b*^ CL (L/h) = 174 × “Ritonavir-CL”.

^*c*^ Ka (h^-1^) = 0.425 × DOSE^-0.127 or -0.138^ × “FOOD-Ka”.

^*d*^ Effect of dose on Ka (simnotrelvir alone).

^*e*^ Effect of dose on Ka (with ritonavir).

^*f*^ F1 = 1 × “Ritonavir-F1” × “FOOD-F1”.

^*g*^ Effect of ritonavir on F1 (administered with the first simnotrelvir dose).

^*h*^ Effect of ritonavir on F1 (administered 12 h before simnotrelvir).

#### Recommendation of starting dose for COVID-19 patients

The simnotrelvir exposures with the dose regimens (300, 450, 600, and 750 mg BID simnotrelvir/100 mg ritonavir) were simulated to predict the efficacious doses. The results (Table S3; Fig. S3 in the Supplement) show that for 300, 450, and 600 mg dose regimens, the percentage of target attainment for Omicron strain EC_90_ (274 ng/mL) at steady state was 52.3%, 75.4%, and 85.6%, respectively. For 750/100 mg BID, the median C_trough_ was 795 ng/mL, and the percentage of target attainment was 90.6%, which was above 90%. This suggests that the 750/100 mg BID simnotrelvir/ritonavir should be adequate for treatment of Omicron strain infection.

### Biomarker confirmation study to investigate dose-exposure-response relationship

**Fig 3:**
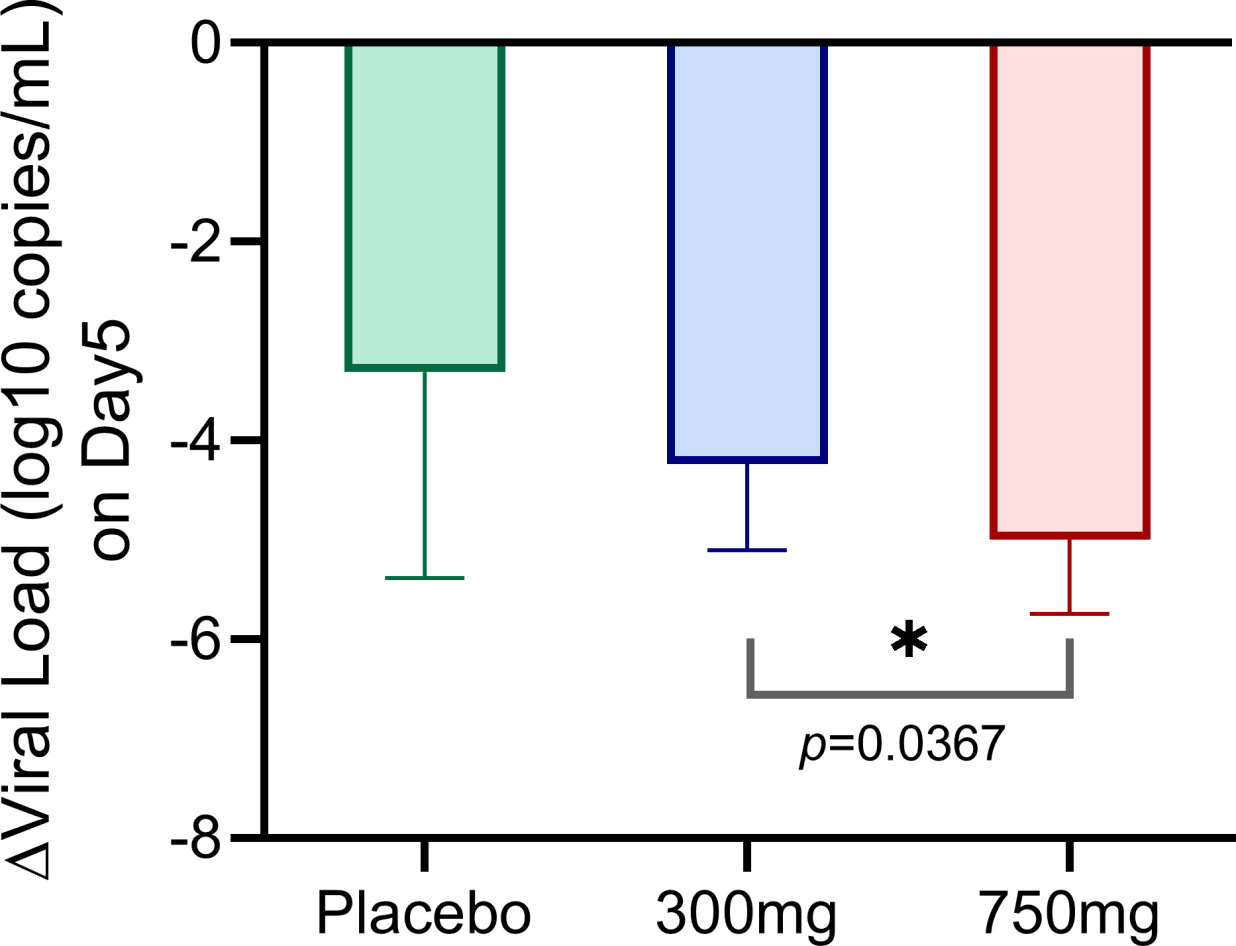
The viral load reduction of different groups in the Phase Ib clinical trial. The viral load reduction data of every group was shown as average and standard deviation (SD).

A total of 32 participants were randomized into high-dose (750/100 mg), low-dose (300/100 mg), or placebo group in Phase Ib, which was conducted in China. One patient in the low-dose group withdrew consent and did not complete the study. The viral load reduction from baseline on day five in the low-dose group was significantly smaller than the high-dose group (−4.236 vs −4.995 log_10_ copies/mL, *P* = 0.0367) (Fig. 3). Similarly, the C_trough_ values were significantly lower in low-dose (514.4 vs 1266.4 ng/mL, *P* = 0.0032). In addition, the viral load reduction in the high-dose group was larger than the placebo group, with a mean difference of −1.69 ± 0.76 log_10_ copies/mL. Moreover, simnotrelvir/ritonavir treatment was well tolerated, and no participants experienced ≥2 grade TEAE (9). Therefore, 750/100 mg simnotrelvir/ritonavir was selected as the predicted therapeutic dose for COVID-19 patients in the Phase II/III study.

Furthermore, the pharmacokinetic prediction of model 1 for patients in Phase Ib performed well (Fig. S4 in the Supplement), with mean prediction error (MPE) and mean absolute prediction (MAPE) values of 17% and 36%, respectively.

### Randomized controlled trial study to confirm benefit-risk ratio

#### Dose regimen confirmation in randomized controlled trial

In the Phase II/III clinical trial in China involving 1,208 patients with mild to moderate COVID-19 who were enrolled within 3 days of symptom onset, 750/100 mg simnotrelvir/ritonavir significantly reduced the time to sustained resolution of COVID-19 symptoms compared with placebo (7.5 days vs 9.0 days, *P* = 0.006). In the subgroup of patients at a high risk of severe illness, this reduction was even more pronounced, with a decrease of approximately 2.5 days. The safety results showed that the incidence of adverse events during treatment was higher in the simnotrelvir group than in the placebo group (29.0% vs 21.6%), and most adverse events were mild or moderate. Notably, no serious adverse events occurred in the simnotrelvir group, whereas serious adverse events were reported in two patients in the placebo group (10).

#### Model update after incorporating patient data

The PopPK model was updated using pharmacokinetic data of all participants who were administered simnotrelvir/ritonavir in Phase Ia, Ib, and II/III trials. A total of 182 participants with 1634 concentrations (Fig. S5 in the Supplement) were enrolled in this PopPK analysis. The median (range) weight and age were 65.0 (39.0–115) kg and 35 (18–67) years, respectively (Table 1). The primary framework of model 2 remained consistent with model 1 (Fig. S6 to S9 in the Supplement). Specific parameter estimates (Tables S4 and S5 in the Supplement) exhibited slight adjustments. The dose simulation results (Fig. S10; Table S6 in the Supplement) showed that 97.9% of participants’ C_trough_ were above the EC_90_ of anti-Omicron. Detailed results for model 2 are provided in the Results in the Supplement.

#### Exposure-response relationship analysis

Ninety-five patients in Phase II/III were included in the exposure-response analysis. The mean predicted AUC and C_max_, and observed C_trough_ were 4,5187.4 ng*h/mL, 6,382.6 ng/mL, and 1,603.6 ng/mL for 750/100 mg (Table S7 in the Supplement).

**Fig 4:**
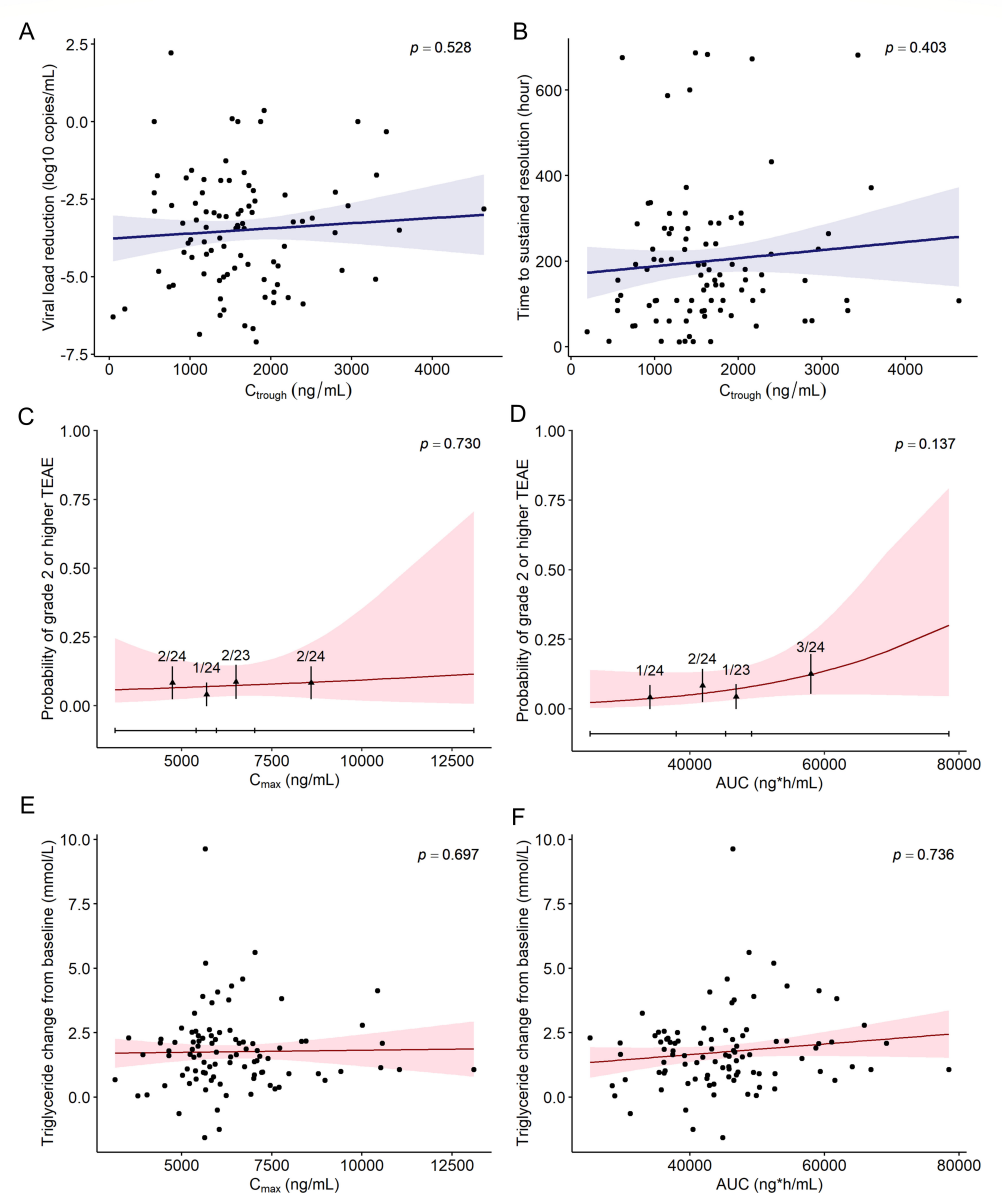
The relationships between efficacy or safety endpoints and simnotrelvir exposure in Phase II/III trials. Efficacy endpoints: (**A**) viral load reduction vs C_trough_; (**B**) time to sustained resolution vs. C_trough_. Safety endpoints: (**C**) grade 2 or higher treatment-emergent adverse event (≥2 grade TEAE) rate vs C_max_; (**D**) ≥ 2 grade TEAE rate vs AUC; (**E**) triglyceride change from baseline vs C_max_; (**F**) triglyceride change from baseline vs. AUC. The fractions in Fig. 4C and D represent the observed number of participants with grade ≥2 TEAEs over the total number of participants within each C_max_ or AUC quartile group.

The linear regression results (Fig. 4A and B; Fig. S11A through D) showed that viral load reduction and time to sustained resolution did not significantly change with simnotrelvir exposures (C_trough_, C_max_ and AUC, all *P* values > 0.05). In addition, the proportions of trough concentrations that were above EC_90_ and 3 times EC_90_ were 97.8% and 87.8%, respectively. These findings confirmed the rationale for using 750/100 mg simnotrelvir/ritonavir to inhibit SARS-CoV-2 viral replication and promote symptom recovery.

In Phase II/III, the rate of ≥2 grade TEAE, including pruritus, constipation, gastric distension, insomnia, hemorrhoids, oral ulcer, and neck rash, was 7.4% (7/95) in the treatment group, which was very close to the placebo group 7.4% (38/511). The logistic regression analysis result showed that the rate of ≥2 grade TEAE would slightly increase with the rise of simnotrelvir exposures (C_max_, C,_trough_ and AUC); however, the changes were not statistically significant (all *P-*values > 0.05) (Fig. 4C and D; Fig. S11E). Besides, as simnotrelvir exposures (C_max_, C,_trough_ and AUC) increased, the relative change in triglyceride levels from baseline on day 5/6 also increased (Fig. 4E and F; Fig. S11F); however, the difference was not statistically significant (*P* > 0.05). These findings suggested that variations in simnotrelvir exposure have a minimal impact on the occurrence of safety events, including elevated triglyceride levels.

Therefore, a comprehensive exposure-response analysis confirmed a favorable benefit-risk ratio for the 750/100 mg simnotrelvir/ritonavir regimen, as it effectively inhibited SARS-CoV-2 viral replication and promoted symptom recovery from Omicron variant infection with minimal impact on safety events.

## DISCUSSION

Using the development of simnotrelvir, an anti-COVID-19 therapeutic, as a case study, this work demonstrates that MIDD effectively accelerated clinical trial process and regulatory approval in China, particularly through optimized dose selection for COVID-19 patients infected with Omicron. The clinical development of simnotrelvir/ritonavir was completed within 9.8 months, comparable to the 10.5-month timeline of nirmatrelvir/ritonavir (11, 12), both benefiting from MIDD-enabled acceleration. The research encompassed three key aspects: a transnational study to predict patients’ starting dose based on first-in-human data, preclinical data, and pharmacodynamic surrogate marker; a biomarker confirmation study to investigate the dose-exposure-response relationship based on viral load data in an early stage clinical trial; and a randomized controlled trial study to confirm the benefit-risk ratio based on the efficacy and safety results and exposure-response relationship using the updated model.

First, the MIDD elucidated the complex disposition of simnotrelvir *in vivo*. Covariate analysis identified several factors. Co-administration with ritonavir influenced simnotrelvir bioavailability by inhibiting CYP3A, which reduced simnotrelvir metabolism (13–15). The simnotrelvir dose amount affected its absorption rate due to low solubility, leading to decreased absorption at higher doses (16). Food intake increased simnotrelvir absorption by enhancing drug solubilization (17), inhibiting intestinal P-glycoprotein, and limiting the simnotrelvir exposure to CYP3A metabolism by “cycling” the drug in and out of enterocytes (18, 19). A positive correlation was observed between simnotrelvir clearance and creatinine clearance (Fig. S6A), as it was excreted via urine with metabolism fully inhibited by ritonavir. The current Phase Ia, Ib, and II/III studies only included individuals with normal renal function or mild renal impairment, and no dose adjustment is required for patients with mild renal impairment (20).

As the COVID-19 pandemic progressed, the Omicron variant became predominant, replacing the Delta variant. Despite generally causing a milder disease, Omicron spreads more rapidly due to multiple mutations in the spike protein that enhance cell entry and immune evasion. Previous drug developments, such as another 3CL^pro^ inhibitor nirmatrelvir, targeted Delta variant, highlighted the urgency to explore the approaches specifically for Omicron. Therefore, when recommending the patients' starting dose for small-scale clinical trials, the EC_90_ specific to Omicron was selected. In the biomarker confirmation stage, the recommended dose (750/100 mg simnotrelvir/ritonavir) showed a significant antiviral advantage over 300/100 mg and placebo. Several studies have suggested that high viral loads are associated with poor clinical outcomes. Increased viral load is a critical factor that triggers an excessive immune response, which can escalate the disease to a more severe state (21), and patients with severe COVID-19 had significantly higher viral loads than those with mild symptoms (22). Thus, in the small patient cohort, the advantages of 750/100 mg simnotrelvir/ritonavir in reducing viral load were crucial for supporting dose selection for larger patient samples in a subsequent Phase II/III trial.

With the prevalence of the Omicron variant, the focus on reducing severe cases is no longer aligned with the current pandemic landscape. In 2022, the NMPA in China revised the primary clinical endpoint of COVID-19 oral medications to the clinical symptom resolution (23, 24). Previously, Paxlovid’s EPIC-SR study, which aimed for symptom resolution, did not meet its primary endpoint (25, 26). Simnotrelvir/ritonavir was the first drug globally to achieve the clinical endpoint of shortening recovery from 11 symptoms. In the Phase II/III trial involving 1,208 symptomatic patients with mild to moderate COVID-19, compared with placebo, simnotrelvir significantly reduced the time to sustained resolution. In investigating the exposure-response relationship, the primary parameters of interest included C_trough_ for efficacy assessment and C_max_ and AUC for safety assessment. In Phase II/III, the response about viral dynamics and clinical symptoms was flat when exposure level fluctuated under the therapeutic dose. These could be attributed to greater than 87.8% of subjects having simnotrelvir concentrations ≥3 times the EC_90_ value for anti-Omicron, with a very limited number of lower concentrations. These results further confirmed that 750/100 mg simnotrelvir/ritonavir was effective against COVID-19. Finally, the safety analysis showed that the ≥2 grade TEAE rate in the treatment group was very close to the placebo group (10). Moreover, the relationships between ≥2 grade TEAE rate or elevated triglycerides and exposure were not statistically significant, suggesting that the safety was reliable in the change of exposure under treatment dose.

Actually, this study has several limitations. While MIDD has been validated for optimizing dosing regimens in the general population with COVID-19, the application of MIDD in special populations, such as pediatric/geriatric patients, those with hepatic/renal impairment, populations of non-East Asian descent, remains unestablished owing to insufficient data. Given the complex involvement of metabolic enzymes and transporters in the disposition of ritonavir and simnotrelvir, potential drug–drug interactions may arise in clinical practice. These will be further investigated in future studies.

In conclusion, by enabling dose selection specifically targeting the Omicron variant, MIDD improved the probability of clinical success and optimized the benefit-risk profile of simnotrelvir, ultimately facilitating its marketing authorization and public availability. This case exemplifies an effective strategy for the rapid development of therapeutics against emerging pathogens during a PHEIC.

## MATERIALS AND METHODS

This study is structured into three main sections, which describe the process of optimizing clinical trial protocols for simnotrelvir based on modeling, as illustrated in Fig. S12.

### Transnational study to predict patients’ starting dose

#### Data from first-in-human clinical trial

The first-in-human trial (Phase Ia) (ClinicalTrials.gov Identifier NCT05339646) (27) included healthy participants treated with simnotrelvir monotherapy and simnotrelvir co-administered with 100 mg ritonavir (simnotrelvir/ritonavir). Healthy participants received a single oral dose of simnotrelvir monotherapy (150–3,000 mg) or simnotrelvir/ritonavir (250/100–1200/100 mg) in single-ascending dose (SAD) part, multiple doses of simnotrelvir monotherapy (750–1,500 mg) or simnotrelvir/ritonavir (150/100–750/100 mg) twice daily (BID) in multiple-ascending dose (MAD) part, and 750/100 mg simnotrelvir/ritonavir in food effect (FE) part (Table S1 in the Supplement). The method for blood sampling protocol, plasma concentration determination, and ethical information are presented in the Methods in the Supplement.

#### PopPK modeling of simnotrelvir

The initial PopPK model (Model 1) was developed using pharmacokinetic data from Phase Ia trial by NONMEM software. Pharmacokinetic parameters and their variability were estimated by first-order conditional estimation method with interactions. The detailed model development, covariate analysis, and model validation are presented in the Methods in the Supplement.

#### Data from preclinical experiment

The antiviral activity of simnotrelvir against SARS-CoV-2 Omicron strain was tested in Vero E6 cells (28). The Omicron strain viral copy numbers were quantified using real-time fluorescence quantitative PCR. The EC_90_ value, which represents the concentration of simnotrelvir at which 90% inhibition of viral replication within cells, was 0.137 µmol/L, suggesting the potent antiviral activity of simnotrelvir against SARS-CoV-2 Omicron. After correction for human plasma unbound fraction (27.5%), the EC_90_ was 274 ng/mL, which was used to optimize the dosing regimen.

#### Patients starting dose recommendation with pharmacodynamic surrogate marker

A starting dose recommendation for patients was based on data from healthy participants in first-in-human trial (Phase Ia), preclinical anti-Omicron activity data, and antiviral pharmacodynamic surrogate marker. The surrogate marker for simnotrelvir pharmacodynamics was simnotrelvir trough concentration (C_trough_) greater than *in vitro* EC_90_ (C_trough_>EC_90_) (29). To achieve maximal antiviral activity, 90% of patients should reach the target C_trough_. Monte Carlo simulations were performed 100 times, and C_trough_ and area under the curve (AUC) were calculated for each participant. The percentage of target achievement was calculated to evaluate each regimen.

### Biomarker confirmation study to investigate dose-exposure-response relationship

Phase Ib (NCT05369676) (30) included COVID-19 patients treated with 300/100 and 750/100 mg simnotrelvir/ritonavir BID (Table S1 in the Supplement). Preliminary validation of the initial dose recommendation was achieved in this small-sample-size patient cohort, focusing on viral load reduction, which was the major pharmacodynamic biomarker. The viral load reductions on day 5 from the baseline of two dose groups were compared using a *t*-test. The rate of grade two or higher treatment-emergent adverse events (≥2 grade TEAE) was also assessed. These findings provide valuable guidance for dose selection in subsequent Phase II/III trials.

Moreover, the predictive performance of Model one on patients’ pharmacokinetic data in Phase Ib was validated (see Methods in the Supplement).

### Randomized controlled trial study to confirm benefit-risk ratio

#### Data from phase II/III clinical trial and PopPK model update of simnotrelvir

The Phase II/III clinical trial (NCT05506176) (10) was a randomized, double-blinded, placebo-controlled study, including a larger sample of COVID-19 patients treated with 750/100 mg simnotrelvir/ritonavir (Table S1 in the Supplement).

When building the final PopPK model of simnotrelvir (Model 2), the pharmacokinetic data from Phase Ib and II/III trials patients were added to modeling data set. Besides, the data of simnotrelvir monotherapy in Phase Ia were removed as simnotrelvir and ritonavir would be co-packaged in the market, and simnotrelvir monotherapy is not considered in clinical practice. The modeling methods were like Model 1 (see Methods in the Supplement).

#### Exposure-response relationship analysis

The exposure-response relationship of simnotrelvir was analyzed using R V4.2.1 (R Foundation for Statistical Computing, Vienna, Austria). For participants with pharmacokinetic data in Phase II/III (10), the efficacy response included viral load reduction and clinical symptoms. The clinical primary endpoint, “time to sustained resolution,” was defined as the time from first administration of treatment until all 11 COVID-19 symptoms were assessed as returning to the absence of COVID-19-related symptoms or restoration to the pre-COVID-19 state (score = 0) and persisted for two consecutive days. The 11 symptoms included cough, nasal congestion or runny nose, sore throat or dry throat, shortness of breath or difficulty in breathing, headache, subjective fever or measured fever, chills or shivering (or feeling cold), muscle or body aches (or soreness), nausea, vomiting, and diarrhea. Furthermore, the safety response included adverse event rate and elevated triglyceride levels, which were the most prevalent adverse event in Phase II/III. The exposure of simnotrelvir was estimated by the final PopPK model (Model 2) using Bayes estimation method.

For efficacy confirmation in a randomized control trial study, the time to sustained resolution was compared between the simnotrelvir and placebo groups with the use of a Peto–Prentice generalized Wilcoxon test. All *P* values are two-sided and were calculated. The relationship between viral load reduction or time to sustained resolution and C_trough_ was analyzed using a linear regression model, which is expressed as


(1) $$E=E_{0}+slope\cdot C_{i}$$

where E_0_ denotes baseline effect, and C_i_ represents drug exposure.

For safety confirmation in a randomized control trial study, linear regression was conducted to assess the relationship between simnotrelvir exposure (C_max_ and AUC) and elevated triglycerides (Equation (1)). Logistic regression was conducted to explore the relationship of exposure and ≥2 grade TEAE rate, which is expressed as


(2) Logit[P(Y=1)]=baseline+slope×exposure

where, Logit() represents the logit transformation of the probability of a specific adverse event occurring in a patient, with the baseline when the patient was not exposed to simnotrelvir.

## Data Availability

Data that support the findings of this study may be available upon reasonable request to the corresponding author.

## SUPPLEMENTAL MATERIAL

Supplemental material aac.00614-25-s0001.docx Fig. S1 to S12; Tables S1 to S7.

ASM does not own the copyrights to Supplemental Material that may be linked to, or accessed through, an article. The authors have granted ASM a non-exclusive, world-wide license to publish the Supplemental Material files. Please contact the corresponding author directly for reuse.
