# Supplementary material for: Model-informed drug development in public health emergency of international concern: accelerating marketing authorization of simnotrelvir
Source: Antimicrob Agents Chemother. 2025 Sep 18;69(11):e00614-25. doi: 10.1128/aac.00614-25 (PMC12587539; doi:10.1128/aac.00614-25)
Supplement: Supplemental material — Fig. S1 to S12; Tables S1 to S7. [file aac.00614-25-s0001.docx]

**Supplementary Information**

**Model-Informed Drug Development in Public Health Emergency of International Concern: Accelerating Marketing Authorization of Simnotrelvir**

Bu-Fan Yao^1#^, Yang Yang^1,3,4#^, Shan-Sen Xu^3,5^, Bo-Hao Tang^1^, Jia Chen^3,4^, Zi-Jia Guo^3,5^, Hong-Lin Hu^3,5^, Wei Zhang^1^, Shu-Meng Fu^1^, Xin-Fang Zhang^1^, Guo-Xiang Hao^1^, Xin-Mei Yang^2^, Lin-Lin Song^2^, Pan-Pan Ye^2^, Lian Liu^3,5^, Shun-Wei Zhu^3,5^, Yi Zheng^1^*, Wei Zhao^1,2^*

# The first two authors contributed equally

* The last two authors contributed equally

1 Department of Clinical Pharmacy, Key Laboratory of Chemical Biology (Ministry of Education), School of Pharmaceutical Sciences, Cheeloo College of Medicine, Shandong University, Jinan, China

2 Department of Clinical Pharmacy, The First Affiliated Hospital of Shandong First Medical University & Shandong Provincial Qianfoshan Hospital, Shandong Engineering and Technology Research Center for Pediatric Drug Development, Shandong Medicine and Health Key Laboratory of Clinical Pharmacy, Jinan, China

3 State Key Laboratory of Neurology and Oncology Drug Development, Nanjing, China

4 Simcere Zaiming Pharmaceutical Co. Ltd., Nanjing, China.

5 Jiangsu Simcere Pharmaceutical Co., Ltd, Nanjing, China

**Corresponding Author:**

Wei Zhao PhD PharmD

Department of Clinical Pharmacy, School of Pharmaceutical Sciences, Shandong University

No.44, Wenhua West Road, Jinan, China

Tel/Fax: +86 531 88383308

**Email**: zhao4wei2@hotmail.com

**eMethods**

The following methods about model building and model validation were applicable to both Model 1 and Model 2.

**Ethical information of simnotrelvir clinical trials**

The inclusion criteria and exclusion criteria of Phase Ia, Phase Ib and Phase II/III could be referred to corresponding published clinical trials [1-3]***.*** These studies were approved by the ethics committee of Shandong Provincial Qianfoshan Hospital (Phase Ia), Shenzhen Third People's Hospital (Phase Ib) and 35 multicenter hospitals including China–Japan Friendship Hospital, Hefei First People's Hospital, Beijing Ditan Hospital Capital Medical University and so on (Phase II/III). They were conducted in accordance with Good Clinical Practice, and the Declaration of Helsinki. All participants provided written informed consent before enrolment.

**Simnotrelvir plasma concentration determination**

The blood sampling protocol for assessing simnotrelvir plasma concentration is detailed in Table S1. Blood samples were promptly centrifuged within 45 minutes after collection, and the resulting plasma was subsequently frozen and stored at temperatures of –20°C or lower. A validated, highly sensitive, and specific high-performance liquid chromatography-tandem mass spectrometry (LC-MS/MS) method was employed to determine the concentration of simnotrelvir in human K2EDTA plasma at WuXi AppTec Co., Ltd (Shanghai, China) for Phase Ia and Teddylab Co., Ltd (Shanghai, China) for Phase Ib and Phase II/III trials. The standard curve spanned a concentration range of 5.00 - 5000 ng/mL and 10-10000ng/mL, respectively. The methods for sample collection and simnotrelvir plasma concentration determination used in subsequent clinical trials will remain consistent with those employed in the Phase Ia study.

**Population pharmacokinetic modelling of simnotrelvir**

Inter-individual variability of the pharmacokinetic parameters was estimated using an exponential model and was expressed as following equation:

θ_i_= θ _mean_*e^ηi^ Equation (1)

where θ _i_ represented the parameter value of the i^th^ subject, θ_mean_ the typical value of the parameter in the population and ηi the variability between subjects which is assumed to follow a normal distribution with a mean of zero and variance ω2.

During forward selection process, the covariates that had potation to influence the pharmacokinetic parameters were all assessed, such as body weight, age, sex, food, dose amount of simnotrelvir, ritonavir co-administration, creatinine clearance. A covariate was included if a significant (p<0.05, χ^2^ distribution with one degree of freedom) decrease (reduction>3.84) in the objective function value (OFV) from the basic model was obtained and a reduction in the variability of the pharmacokinetic parameter. Then, all the significant covariates were simultaneously added into a ‘full’ model. And then after that, each covariate was independently removed from the ‘full’ model. When the OFV increased more than 6.635 (p<0.01, χ^2^ distribution), the covariate was regarded as significantly correlated with the pharmacokinetic parameter and therefore it was retained in the final model.

**Model validation**

***(1) Internal model validation***

Goodness-of-fit plots, including population prediction (PRED) *versus* observed (DV); individual prediction (IPRED) *versus* DV; conditional weighted residuals (CWRES) *versus* time and CWRES *versus* PRED were initially used to validate the model[4]. A nonparametric bootstrap with re-sampling and replacement was conducted to assess the stability and performance of the final model. Re-sampling was repeated 1000 times and the values of parameters estimated from the bootstrap procedure were compared with those estimated from the original data set. The entire procedure was performed in an automated way, using PsN (v2.30) [5]. The final model was also validated graphically and statistically using normalized prediction distribution errors (NPDE). One thousand datasets were simulated with the final population model parameters. NPDE results were provided graphically by the NPDE R package (v1.2) [6]: (i) QQ-plot of the NPDE; (ii) histogram of the NPDE. The NPDE is expected to follow the standard normal distribution. Lastly, the final model was also evaluated by visual predictive checks (VPC) to assess its predictive performance. Using the estimated parameters from the final model, 1000 simulations were performed to obtain the 5th, 50th, and 95th percentiles of the predicted values, creating confidence intervals of these prediction percentiles. The model was considered satisfactory if 90% of the observed data fell within the 90% prediction intervals and showed a uniform distribution around the mean.

***(2) External model validation***

In order to validate the model, the independent dataset was obtained and the same opportunistic PK sampling design was performed. Information of patients for external validation was available which included basic physiological information, dosing information, sampling information and covariate information.

Mean absolute prediction (MAE) and mean prediction error (MPE) were applied to calculate bias and imprecision of predictive performance [7] (Eqs. 2 and 3). In addition, the number of patients with MAE and MPE within the range of ±30% were calculated [8].

 Equation (2)

 Equation (3)

**eResults**

**Model 1 development**

***Model building***

A total of 72 healthy volunteers from the first-in-human clinical trial (Phase Ia) were included in population pharmacokinetic analysis. The median (minimum-maximum) weight and age were 64.4 (46.1–82.9) kg and 30 (20–50) years, respectively (Table 1). One thousand seven hundred and forty-two concentrations were available to build the population pharmacokinetic model. The concentrations of simnotrelvir ranged from below the limit of quantification (BLQ) to 12100 ng/mL. For every subject, the first concentration below the lower limit of quantification remained. There were total of 95 concentrations accounted for 5.5% and they were replaced by 2.5 ng/mL which was half of LOQ. The ln concentration versus time curve were shown in Figure 2A-D.

In the basic model building, the two-compartment model was more stable and lower objective function value (OFV, 23016.833) compared with one-compartment model (OFV 26044.814). Thus, a two-compartment model with first-order absorption described the data best. The pharmacokinetic parameters derived from the model included absorption rate constant (Ka), clearance (CL), central volume of distribution (V2), and peripheral volume of distribution (V3), intercompartment clearance (Q), relative bioavailability (F1), absorption lag time (ALAG) of simnotrelvir. The exponential model best described the inter-individual variability. The inter-individual variability was estimated for CL, V2 and Ka. Residual variability was best described using a combined proportional and additive residual error model.

***Covariate analysis***

For that ritonavir showed time-dependent inhibition and competitive inhibition of CYP3A4, the combined use of ritonavir showed a significant effect on CL (ΔOFV -1,057.568) and F1 (ΔOFV -26.761) compared with simnotrelvir monotherapy. Simnotrelvir dose amount showed a significant effect on Ka, which caused 21.827 points drop in OFV. Food status (fasted or fed) could apparently influence the absorption parameters (Ka and F1) and the OFV decreased 77.615 points. Finally, the CL was significantly affected by creatinine clearance (ΔOFV -4.295). The body weight, age and sex did not significantly affect the key pharmacokinetic parameters (CL, V2, V3), for that all the reduction of OFV were less than 3.84. In the backward selection process, the influence of creatinine clearance on CL was removed for that it was not significant (ΔOFV > 6.63). In addition, inter-occasion variability was estimated for V2 to describe the variability of PK characteristic between first and second intensive plasma sampling, and this could decrease OFV by 68.280 points. A detailed covariate analysis process was shown in Table S2.

The parameter estimated values of the final model were summarized in Table 2. The median (range) of estimated CL and Ka were 150.52 (112.90-397.93) L/h and 0.19 (0.10-0.33) h^-1^, respectively when simnotrelvir was administrated alone. Besides, the median (range) of estimated CL and Ka were 39.05 (26.27-62.16) L/h and 0.20 (0.11-0.29) h^-1^, respectively when simnotrelvir was co-administrated with ritonavir.

The covariates results showed that the relative bioavailability was increased by 39%, and absorption rate constant was increased by 49% in fed status compared with fasted status. The relative bioavailability could also be increased by 61% or 36% when ritonavir was administrated 12 hours prior to simnotrelvir or administrated simultaneously, compared with simnotrelvir monotherapy. The absorption rate constant was decreased with the increase of simnotrelvir dose amount which could explain the reason of non-linear characteristic of simnotrelvir dose-exposure relationships to some extent, and the exponent was -0.127 and -0.138 for simnotrelvir monotherapy and simnotrelvir/ritonavir co-administration, respectively.

***Model internal evaluation***

Model diagnostics confirmed the acceptability of the final simnotrelvir model prediction performance. Figure 3A-B illustrated the absence of any systematic bias in the predictions. Additionally, Figure 3C-D revealed the absence of discernible trends in the diagnostic plots of conditional weighted residuals (CWRES) versus time and population prediction (PRED). Furthermore, the median parameter estimates obtained from the bootstrap analysis were consistent with the corresponding values derived from the final model, suggesting the stability of the final model for re-estimating population pharmacokinetic parameters (Table 2). As depicted in Figure S1, the NPDE distribution and histogram closely matched the standard normal distribution and density, indicative of a good fit of the model to the individual data. The mean and variance of NPDE were 0.00606 and 0.936, respectively. Finally, the visual predictive checks (VPC) analysis demonstrated a strong performance of the NONMEM model, as evidenced by the close alignment between model-simulated data and observed data (as shown in Figure S2).

***Model external validation***

The external validation data from 24 COVID-19 patients were obtained in Phase Ib clinical trial. All of them met the inclusion and exclusion criteria and the informed consent was obtained. The median (minimum-maximum) values of body weight and age were 68.0 (50.0-100) kg and 24.2 (17.3-29.2) years, respectively. Patient characteristics of external validation data were shown in Table 1. The mean prediction error (MPE) and mean absolute prediction (MAPE) values were 17% and 36%, respectively. The patients within the range of ± 30% of MPE were account for 75.9%. The goodness of fit plots of external validation also showed good performance of prediction in Phase Ib subjects’ exposure (Figure S4).

**Model2 development**

***Model building***

A total of 182 subjects were enrolled in this population pharmacokinetic analysis (Phase Ia 60 healthy volunteers, Phase Ib 24 COVID-19 patients, Phase II/III 98 COVID-19 patients, respectively). All subjects met the inclusion and exclusion criteria, and informed consents were obtained. The mean (standard deviation) values of body weight and age were 64.7 (10.6) kg and 36.9 (12.5) years, respectively. Subjects’ characteristics are summarized in Table 1.

One thousand six hundred and thirty-four concentrations were available to build the population pharmacokinetic model. The concentrations of simnotrelvir ranged from lower than the limit of quantification (LOQ) to 17252 ng/mL. For every subject, the first concentration below the lower limit of quantification remained. There were total of 87 concentrations accounted for 5.3% and they were replaced by 2.5 ng/mL which was half of LOQ. The ln concentration versus time curve were shown in Figure S5A-D.

In the basic model building, the two-compartment model was more stable and lower objective function value (OFV, 21839.888) compared with one-compartment model (OFV 22296.973). Thus, a two-compartment model with first-order absorption described the data best. The pharmacokinetic parameters derived from the model included absorption rate constant (Ka), clearance (CL), central volume of distribution (V2), and peripheral volume of distribution (V3), intercompartment clearance (Q), relative bioavailability (F1), absorption lag time (ALAG) of simnotrelvir. The exponential model best described the inter-individual variability. The inter-individual variability was estimated for CL, V2 and Ka. Residual variability was best described using a combined proportional and additive residual error model.

***Covariate analysis***

For that ritonavir showed time-dependent inhibition and competitive inhibition of CYP3A4, the relative administration time of ritonavir compared with simnotrelvir (whether ritonavir was administrated with simnotrelvir simultaneously or was 12 hours prior to simnotrelvir) showed a significant effect on F1 (OFV decreased 22.425 points). Simnotrelvir dose amount showed a significant effect on Ka, which caused 28.375 points drop in OFV. Food status (fasted or fed) could apparently influence the absorption parameters (Ka and F1) and the OFV decreased 283.425 points. Finally, the CL was significantly affected by creatinine clearance (ΔOFV -10.949). In addition, inter-occasion variability was estimated for V2 to describe the variability of PK characteristic between first and second intensive plasma sampling, and this could decrease OFV by 122.324 points. A detailed covariate analysis process was shown in Table S4.

The parameter estimated values of the final model were summarized in Table S5. The median (range) of estimated CL and Ka were 22.12 (11.09-42.12) L/h and 0.17 (0.11-0.28) h^-1^, respectively when simnotrelvir was co-administrated with ritonavir.

The covariates results showed that the relative bioavailability was increased by 36%, and absorption rate constant was increased by 51% in fed status compared with fasted status. The relative bioavailability could also be increased by 15% when ritonavir was administrated 12 hours prior to simnotrelvir verses administrated simultaneously. The clearance of simnotrelvir decreased with increased creatinine clearance (CRCL) in adults, and the relationship of simnotrelvir CL with CRCL was shown in Figure S6A. The absorption rate constant was decreased with the increase of simnotrelvir dose amount which could explain the reason of non-linear characteristic of simnotrelvir dose-exposure relationships to some extent (shown as Figure S6B).

***Model evaluation***

Model diagnostics confirmed the acceptability of the final simnotrelvir model prediction performance. Figure S7A-B illustrated the absence of any systematic bias in the predictions. Additionally, Figure S7C-D revealed the absence of discernible trends in the diagnostic plots of conditional weighted residuals (CWRES) versus time and population prediction (PRED). Furthermore, the median parameter estimates obtained from the bootstrap analysis were consistent with the corresponding values derived from the final model, suggesting the stability of the final model for re-estimating population pharmacokinetic parameters (Table S5). As depicted in Figure S8, the NPDE distribution and histogram closely matched the standard normal distribution and density, indicative of a good fit of the model to the individual data. The mean and variance of NPDE were 0.00295 and 0.787, respectively. Finally, the visual predictive checks (VPC) analysis demonstrated a strong performance of the NONMEM model, as evidenced by the close alignment between model-simulated data and observed data (as shown in Figure S9).

Figures and Tables in the supplementary information


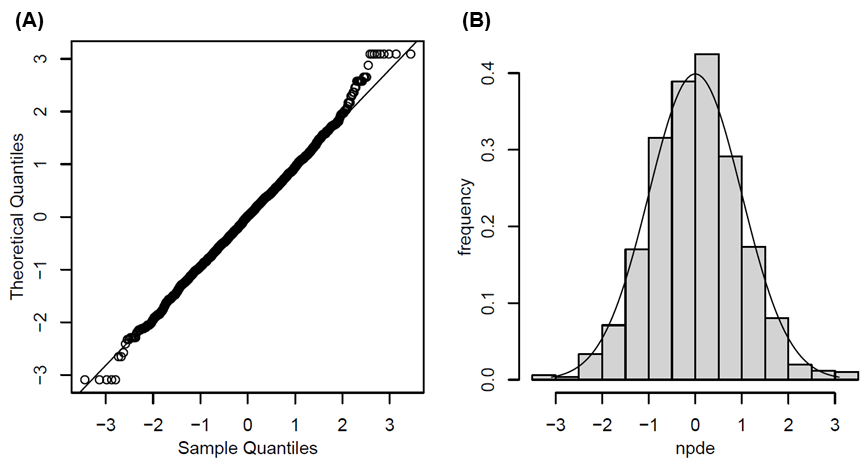


Figure S**1. Normalized prediction distribution errors plots (NPDE) of final simnotrelvir Model 1.** (A) Theoretical quantiles versus sample quantiles; (B) NPDE distribution histogram.


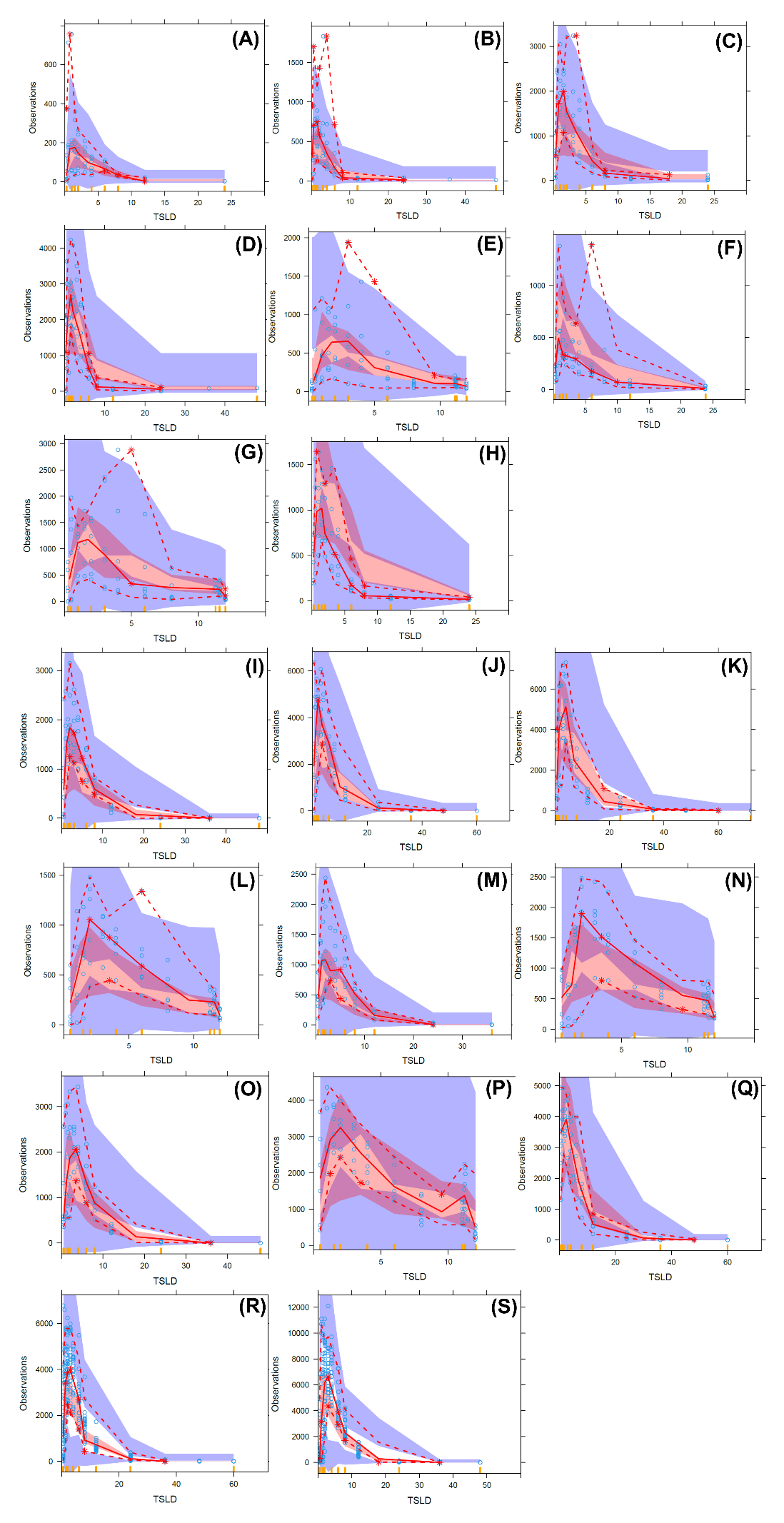


Figure S2. Visual predictive checks (VPC) results of final simnotrelvir Model 1, including simnotrelvir monotherapy and simnotrelvir/ritonavir combination therapy. Simnotrelvir monotherapy: (A) 150 mg single dose (SD); (B) 500 mg SD; (C) 1500 mg SD; (D) 3000 mg SD; (E) 750 mg twice a day (BID) after first dose; (F) 750 mg BID at steady state; (G) 1500 mg BID after first dose; (H) 1500 mg BID at steady state. Simnotrelvir/ritonavir combination therapy: (I) 250/100 mg SD; (J) 750/100 mg SD; (K) 1200/100 mg SD; (L) 150/100 mg BID after first dose; (M) 150/100 mg BID at steady state; (N) 300/100 mg BID after first dose; (O) 300/100 mg BID at steady state; (P) 750/100 mg BID after first dose; (Q) 750/100 mg BID at steady state; (R) 750/100 mg SD fasted; (S) 750/100 mg SD fed. All the groups were at fasted state except for “(S) 750/100 mg SD fed”.


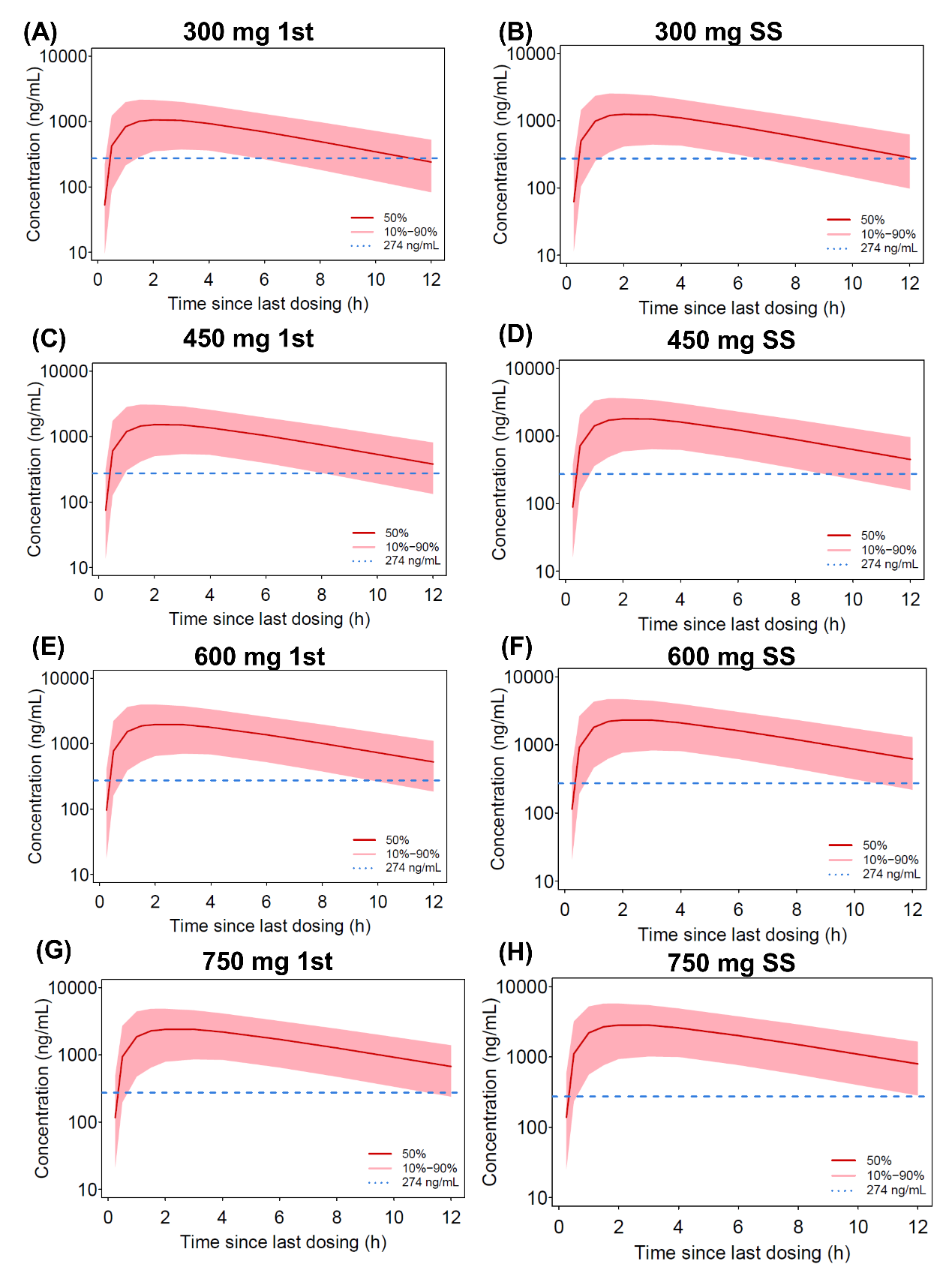


Figure S**3. Dose simulation results of different simnotrelvir/ritonavir dose regimens twice daily using Model 1.** (A) 300/100 mg twice daily (BID) after first dose; (B) 300/100 mg BID at steady state; (C) 450/100 mg BID after first dose; (D) 450/100 mg BID at steady state; (E) 600/100 mg BID after first dose; (F) 600/100 mg BID at steady state; (G) 750/100 mg BID after first dose; (H) 750/100 mg BID at steady state. 1st, after first dose; SS, steady state.


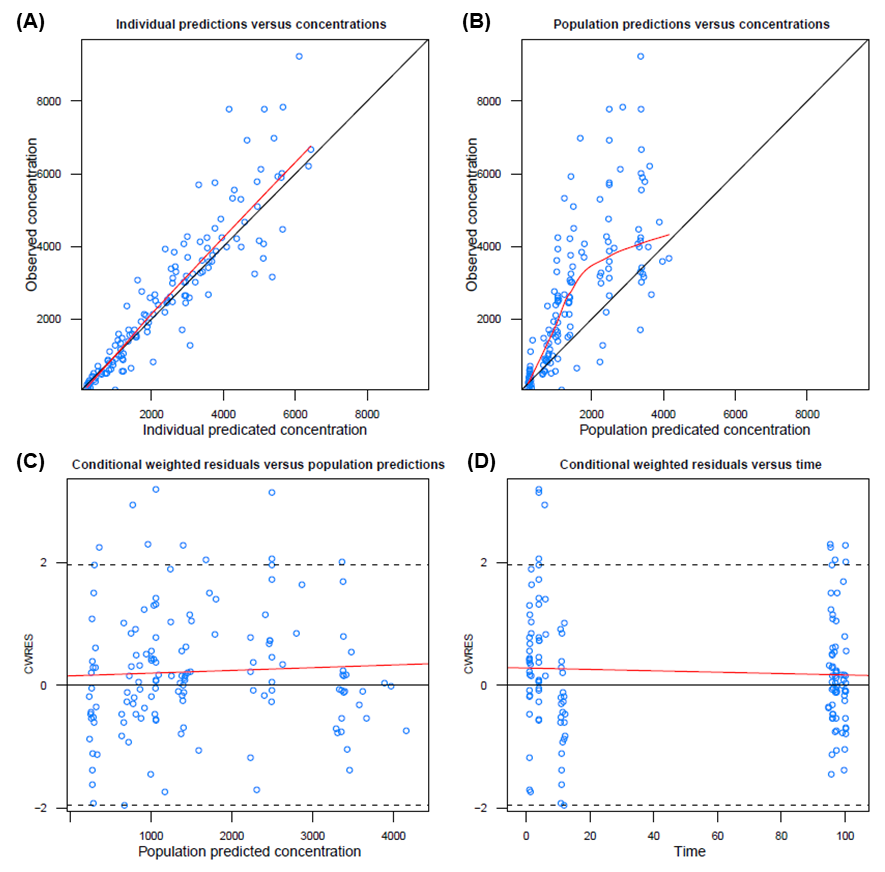


Figure S4. Goodness of fit plots of final simnotrelvir Model 1 for external validation using Phase Ib dataset. (A) Observed concentration versus individual predicated concentration; (B) Observed concentration versus population predicated concentration; (C) Conditional weighted residuals versus population predicated concentration; (D) Conditional weighted residuals versus time.


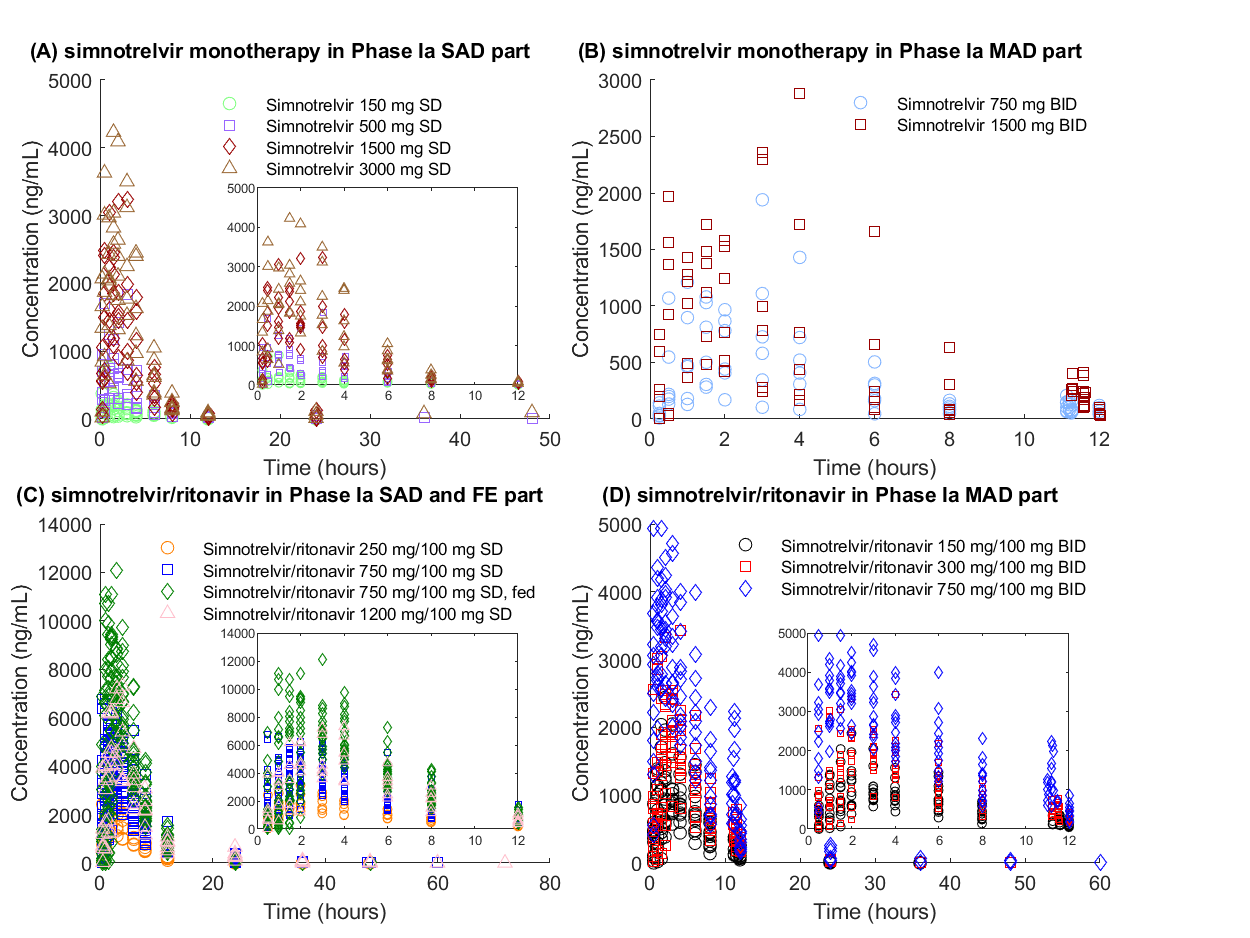


Figure S5. The concentration versus time curve of simnotrelvir used in Model 2. (A) Single-ascending dose part in Phase Ia; (B) Multiple-ascending dose part in Phase Ia; (C) Phase Ib; (D) Phase II/III.


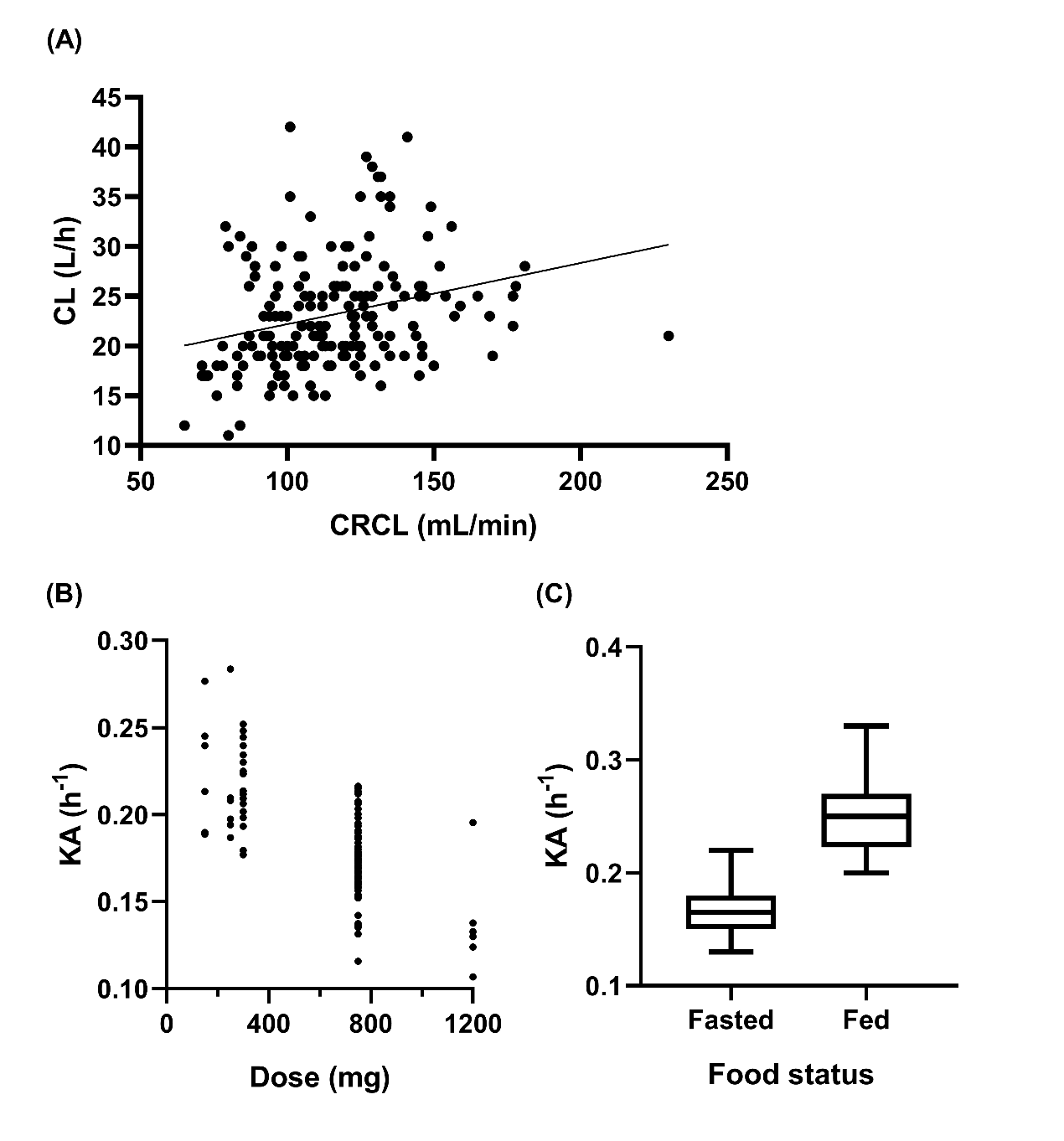


Figure S6. The relationship between covariates and pharmacokinetic parameters obtained from Model 2. (A) clearance (CL) and serum creatinine clearance (CRCL); (B) absorption rate constant and dose; (C) absorption rate constant and food status.


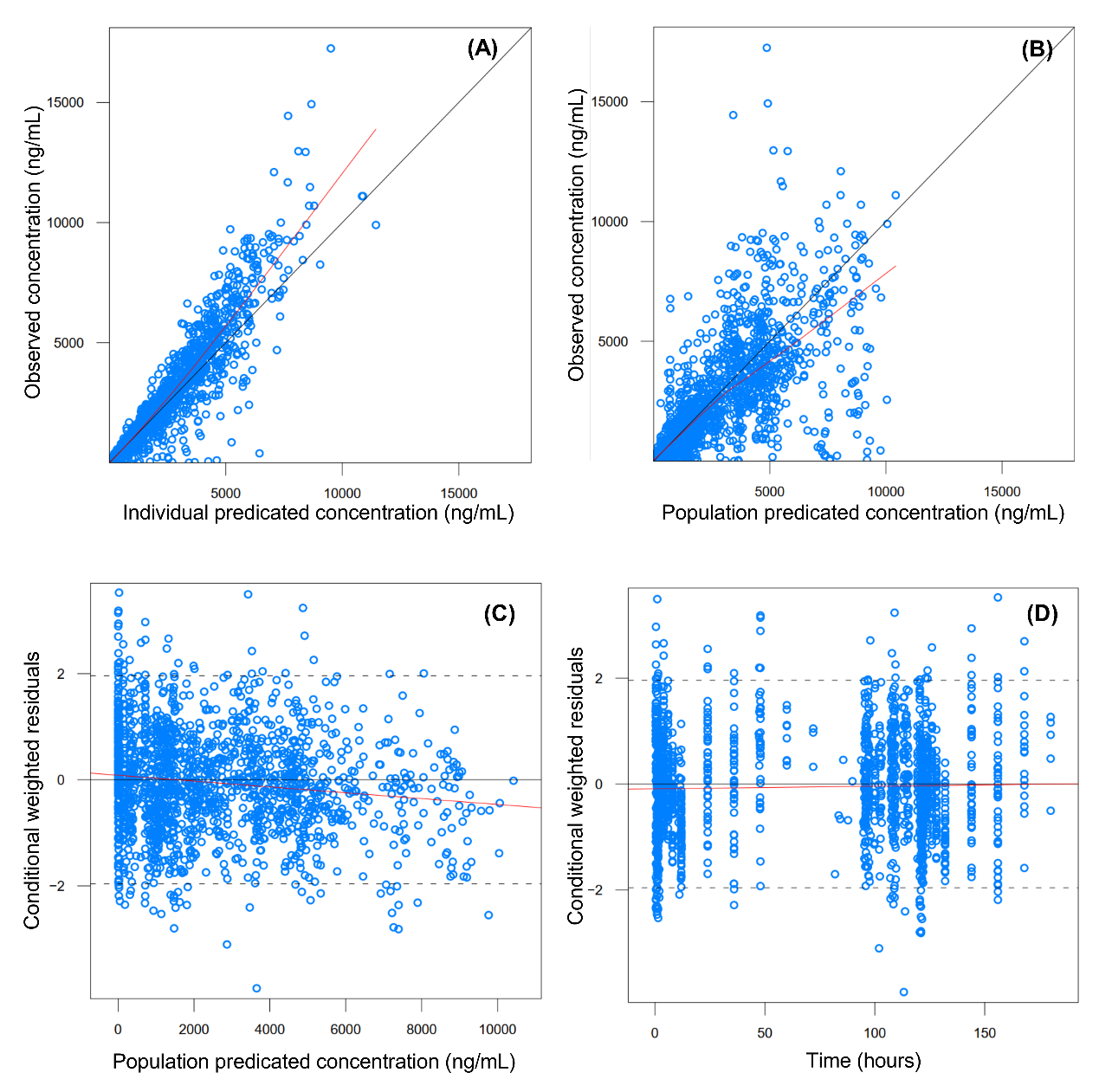


Figure S7. Goodness of fit plots of final simnotrelvir Model 2. (A) Observed concentration versus individual predicated concentration; (B) Observed concentration versus population predicated concentration; (C) Conditional weighted residuals versus population predicated concentration; (D) Conditional weighted residuals versus time.


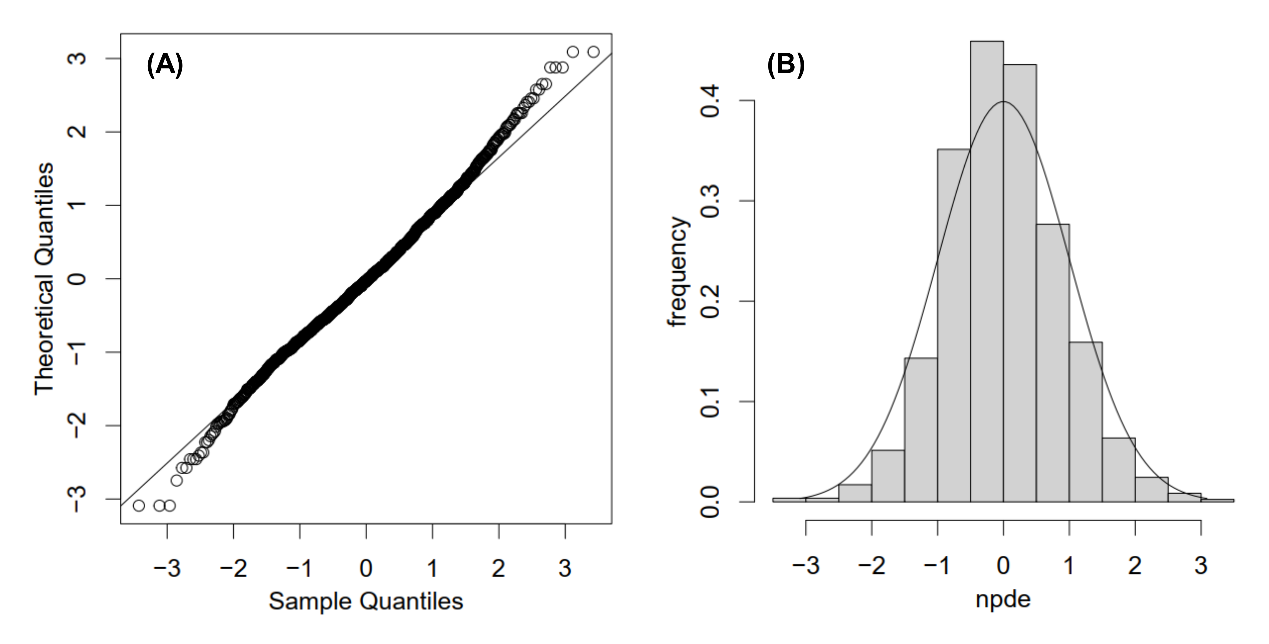


Figure S8. Normalized prediction distribution errors plots (NPDE) of final simnotrelvir Model 2. (A) Theoretical quantiles versus sample quantiles; (B) NPDE distribution histogram.


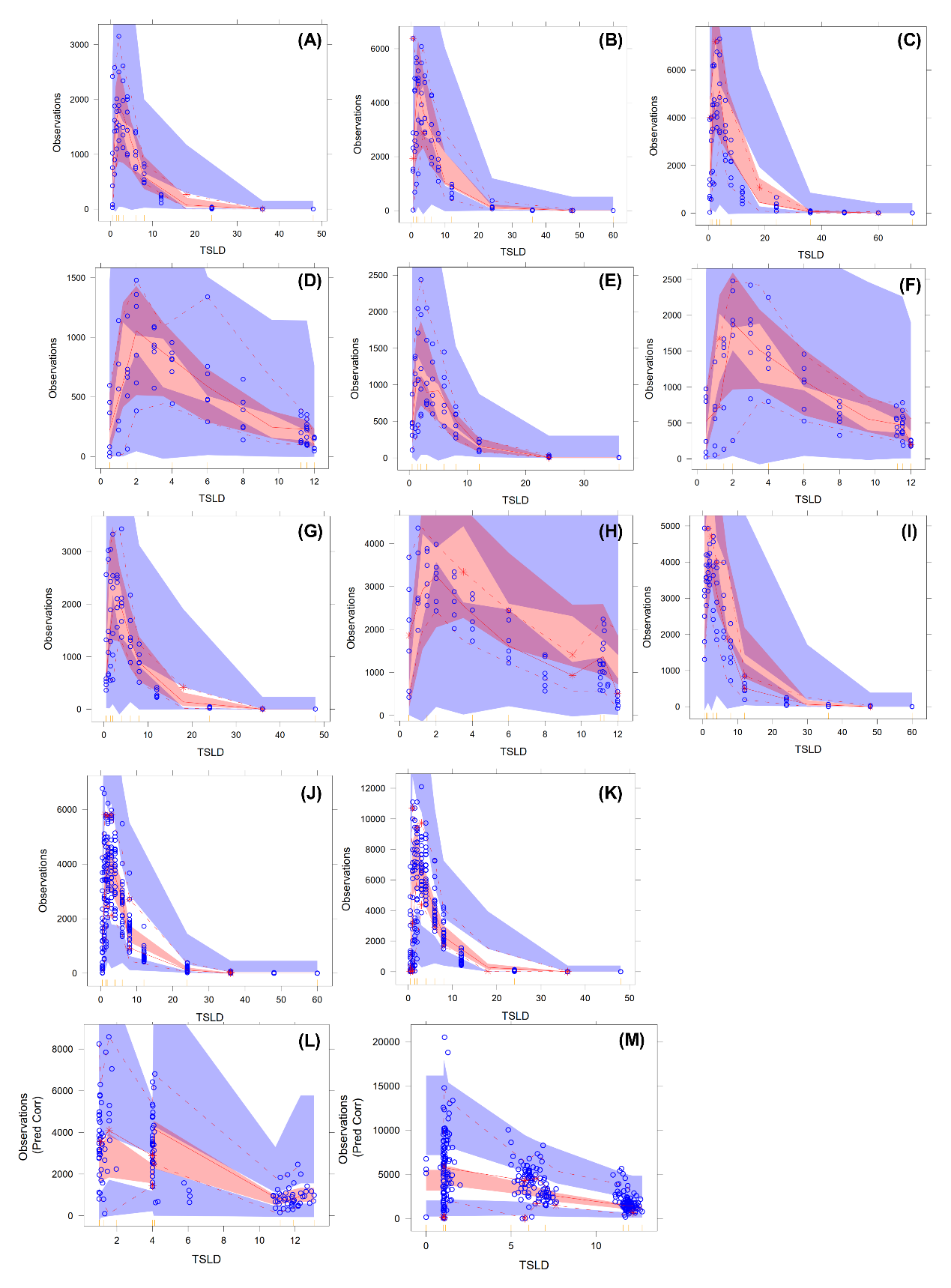


Figure S**9. Visual predictive checks (VPC) results of final simnotrelvir Model 2.** (A) 250 mg single dose (SD); (B) 750 mg SD; (C) 1200 mg SD; (D) 150 mg twice a day (BID) after first dose; (E) 150 mg BID at steady state; (F) 300 mg BID after first dose; (G) 300 mg BID at steady state; (H) 750 mg BID after first dose; (I) 750 mg BID at steady state; (J) 750 mg SD fasted; (K) 750 mg SD fed; (L) Phase Ib (300 mg and 750 mg BID); (M) Phase II/III 750 mg BID. All the groups were at fasted state except for “(K) 750 mg SD fed”.


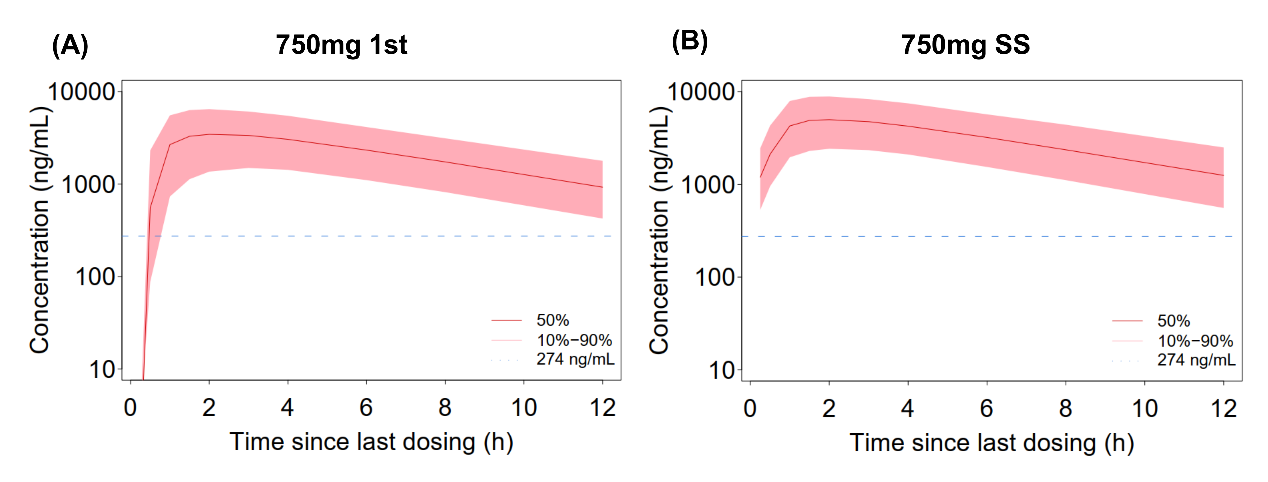


Figure S10. Dose simulation results of 750 mg BID using Model 2. 1st, after first dose; SS, steady state.


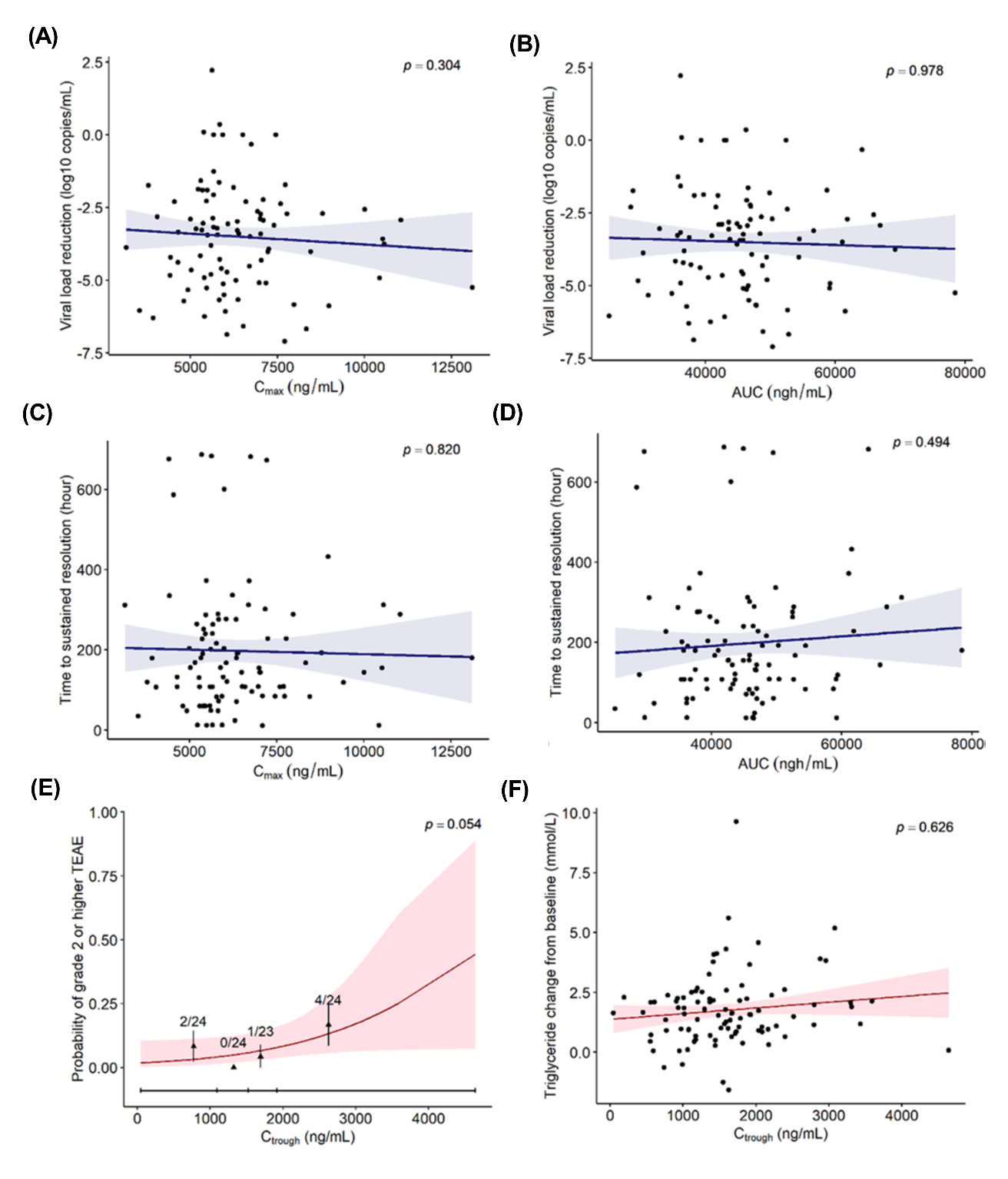


**Figure S11. The relationships between efficacy or safety endpoints and simnotrelvir exposure in Phase II/III trial. Efficacy endpoints:** A. viral load reduction vs. C_max_; B. viral load reduction vs. AUC; C. time to sustained resolution vs. C_max_; D. time to sustained resolution vs. AUC. **Safety endpoints:** E. grade 2 or higher treatment-emergent adverse event (≥2 grade TEAE) rate vs. C_trough_; F. triglyceride change from baseline vs. C_trough_. The fractions in Figure 4E represent the observed number of participants with grade ≥2 TEAEs over the total number of participants within each C_trough_ quartile group.

**
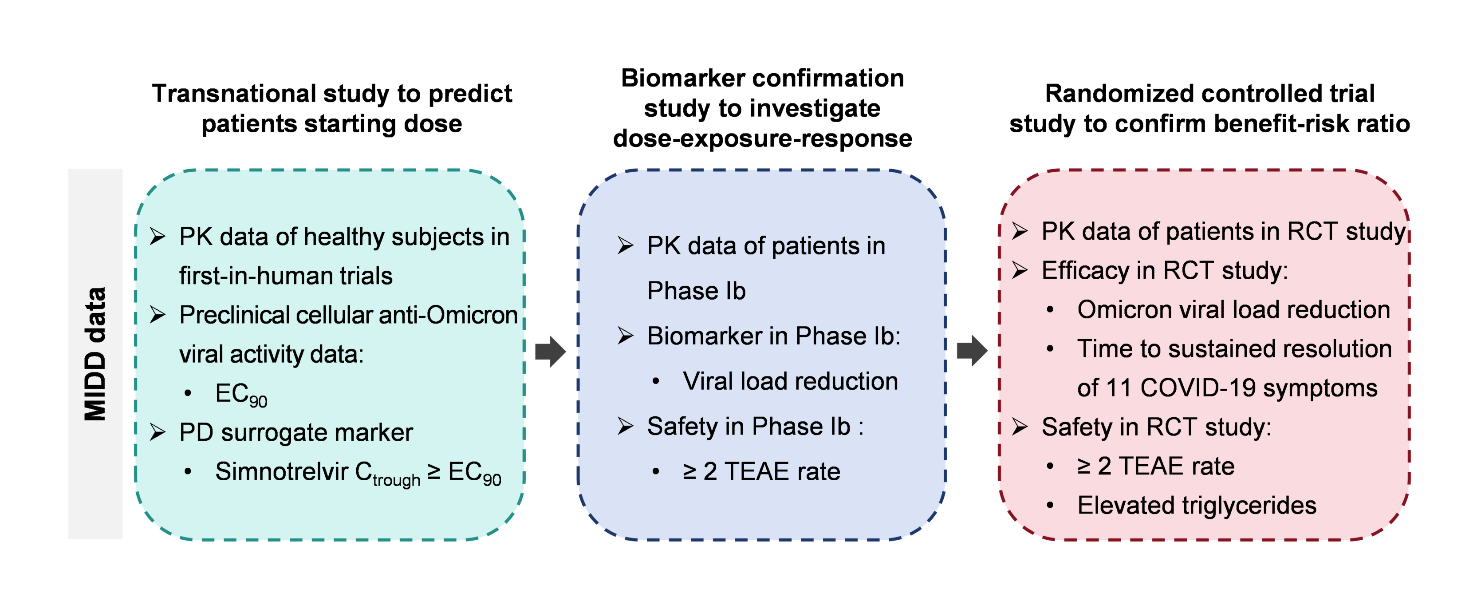
Figure S12. The flowchart of this model-informed drug development study.**

**Table S1. Dose regimens and sampling time of each clinical trials included in the Model 1 and Model 2 PopPK analysis.**

| **Study ID** | **Participants** | **Simnotrelvir dose (mg)** | **Ritonavirdose (mg)** | **Frequency** | **Food status** | **Sampling time** | **Model 1 building** | **Model 2 building** |
| --- | --- | --- | --- | --- | --- | --- | --- | --- |
| Phase Ia-SAD, without ritonavir | Healthy volunteers | 150, 500, 1500, 3000 | 0 | Single dose | Fasted | Intensive blood sampling after single dose | Yes | No |
| Phase Ia-MAD, without ritonavir | Healthy volunteers | 750, 1500 | 0 | Twice a day | Fasted | Intensive blood sampling after first dose and last dose, trough concentration sampling after 4th, 8th,10th dose | Yes | No |
| Phase Ia-SAD, with ritonavir | Healthy volunteers | 250, 750, 1200 | 100 | Single dose | Fasted | Intensive blood sampling after single dose | Yes | Yes |
| Phase Ia-MAD, with ritonavir | Healthy volunteers | 150, 300, 750 | 100 | Twice a day | Fasted | Intensive blood sampling after first dose and last dose, trough concentration sampling after 4th, 8th,10th dose | Yes | Yes |
| Phase Ia-FE | Healthy volunteers | 750 | 100 | Single dose | Fasted or fed | Intensive blood sampling after single dose | Yes | Yes |
| Phase Ib | COVID-19 patients | 300, 750 | 100 | Twice a day | Fasted | Three blood collections at first dose and steady state | No | Yes |
| Phase II/III | COVID-19 patients | 750 | 100 | Twice a day | Fasted | Three blood collections at steady state | No | Yes |

SAD, single-ascending dose part; MAD, multiple-ascending dose part; FE, food effect part.

Table S2. Covariate analysis of Model 1.

| **Run ID** | **Description** | **OFV** | **ΔOFV** |
| --- | --- | --- | --- |
| **Forward selection process** | | | |
| 1 | Basic model | 23016.833 | - |
| 2 | Run 1 + Ritonavir to CL ^a^ | 21959.265 | -1,057.568 |
| 3 | Run 2 + Ritonavir to F1 ^b^ | 21932.504 | -26.761 |
| 4 | Run 3 + Simnotrelvir dose amount to Ka | 21910.677 | -21.827 |
| 5 | Run 4 + Food to F1 | 21836.388 | -74.289 |
| 6 | Run 4 + Food to Ka | 21738.790 | -171.887 |
| 7 | Run 6 + Food to F1 | 21661.175 | -77.615 |
| 8 | Run 6 + CLCR to CL | 21656.880 | -4.295 |
| **Full model——Run 8** | | | |
| **Backward selection process** | | | |
| 9 | Run 8 - Ritonavir to CL ^a^ | 21706.646 | 49.766 |
| 10 | Run 8 - Ritonavir to F1 ^b^ | 21683.536 | 26.656 |
| 11 | Run 8 - Simnotrelvir dose amount to Ka | 21686.884 | 30.004 |
| 12 | Run 8 - Food to F1 | 21732.204 | 75.324 |
| 13 | Run 8 - Food to Ka | 21830.805 | 173.925 |
| 14 | Run 8 - CLCR to CL | 21661.175 | 4.295 |
| **Inter-occasion variability** | | | |
| 15 | Run 7 + Inter-occasion variability to V2 | 21592.895 | -68.280 |
| **Final Model——Run 15** | | | |

OFV, objective function value; F1, relative bioavailability; Ka, absorption rate constant; CL, clearance; CLCR, creatinine clearance rate; V2, central volume of distribution.

^a^, Effect of co-administration ritonavir to CL.

^b^, Effect of relative administration time of ritonavir on F1 (Administration time of ritonavir was before simnotrelvir, compared with simultaneous administration).

Table S3. Dose simulation result of simnotrelvir/ritonavir in fasted status after Model1.

|  | **300/100 mg BID** | |  | **450/100 mg BID** | |  | **600/100 mg BID** | |  | **750 mg BID** | |
| --- | --- | --- | --- | --- | --- | --- | --- | --- | --- | --- | --- |
|  | First dose | Steady state |  | First dose | Steady state |  | First dose | Steady state |  | First dose | Steady state |
| C_trough_ (ng/mL), median (10^th^-90^th^) | 240 (83-526) | 284 (98-622) |  | 379 (133-810) | 449 (158-959) |  | 525 (185-1099) | 622 (220-1302) |  | 672 (239-1389) | 795 (283-1645) |
| AUC_0-12_ (ng*h/mL), median (10^th^-90^th^) | 8098 (5237-12339) | 9587 (6202-14607) |  | 11934 (7714-18215) | 14129 (9134-21565) |  | 15697 (10137-23984) | 18583 (12001-28390) |  | 19394 (12526-29723) | 22959 (14829-35186) |
| %Subjects C_trough_>EC_90_^a^ | 42.2 | 52.3 |  | 68.3 | 75.4 |  | 81 | 85.6 |  | 87.2 | 90.6 |

Note: ^a^ The EC_90_ for the simnotrelvir against the Omicron virus was 274 ng/mL.

Table S4. Covariate analysis of Model 2.

| **Run ID** | **Description** | **OFV** | **ΔOFV** |
| --- | --- | --- | --- |
| **Forward selection process** | | | |
| 1 | Basic model | 22307.386 | - |
| 2 | Run 1 + Ritonavir to F1 ^a^ | 22284.961 | -22.425 |
| 3 | Run 2 + Simnotrelvir dose amount to Ka | 22256.586 | -28.375 |
| 4 | Run 3 + Food to F1 | 22183.531 | -73.055 |
| 5 | Run 3 + Food to Ka | 22045.62 | -210.966 |
| 6 | Run 5 + Food to F1 | 21973.161 | -72.459 |
| 7 | Run 6 + CLCR to CL | 21962.212 | -10.949 |
| **Full model——Model 7** | | | |
| **Backward selection process** | | | |
| 8 | Model 7 - Food to F1 | 22037.366 | +75.154 |
| 9 | Model 7 - Food to Ka | 22173.792 | +211.58 |
| 10 | Model 7 - Simnotrelvir dose amount to Ka | 22031.337 | +69.125 |
| 11 | Model 7 - Ritonavir to F1 ^a^ | 21984.503 | +22.291 |
| 12 | Model 7 - CLCR to CL | 21973.161 | +10.949 |
| **Inter-occasion variability** | | | |
| 13 | Model 7 + Inter-occasion variability to V2 | 21839.888 | -122.324 |
| **Final Model——Model 13** | | | |

^a^ , Effect of relative administration time of ritonavir on F1 (Administration time of ritonavir was before simnotrelvir, compared with simultaneous administration).

OFV, objective function value; F1, relative bioavailability; Ka, absorption rate constant; CL, clearance; CLCR, creatinine clearance rate; V2, central volume of distribution.

Table S5. Population pharmacokinetic parameters of simnotrelvir and bootstrap results of Model 2.

| **Parameters** | **Final Model** | |  | **Bootstrap (n=1000)** | | |
| --- | --- | --- | --- | --- | --- | --- |
|  | **Estimate** | **RSE%** |  | **Median** | **5^th^** | **95^th^** |
| CL (L/h) ^a^ | 22.6 | 4.3 |  | 22.6 | 20.9 | 24.2 |
| CRCL-CL | 0.39 | 26.7 |  | 0.388 | 0.213 | 0.568 |
| V2 (L) | 9.48 | 21.0 |  | 9.30 | 4.91 | 12.9 |
| V3 (L) | 3.92 | 14.7 |  | 3.95 | 1.82 | 6.91 |
| Q (L/h) | 17.4 | 14.4 |  | 17.5 | 9.45 | 26.3 |
| Ka (h^-1^)^b^ | 0.755 | 23.7 |  | 0.743 | 0.523 | 1.12 |
| DOSE-Ka | -0.229 | 16.2 |  | -0.228 | -0.288 | -0.172 |
| FOOD-Ka | 1.51 | 3.6 |  | 1.51 | 1.42 | 1.60 |
| F1^c^ | 1 | 0 |  | 1 | 1 | 1 |
| FOOD-F1 | 1.36 | 3.0 |  | 1.36 | 1.29 | 1.43 |
| Ritonavir-F1 | 1.15 | 3.9 |  | 1.15 | 1.07 | 1.23 |
| ALAG (h) | 0.447 | 3.4 |  | 0.448 | 0.419 | 0.483 |
| **Inter-individual variability (CV%)** | | | | | | |
| CL | 27.0 | 6.3 |  | 26.6 | 23.9 | 29.9 |
| V2 | 81.6 | 28.4 |  | 82.4 | 42.7 | 114 |
| Ka | 16.0 | 7.5 |  | 15.7 | 13.6 | 17.5 |
| **Inter-occasion variability (CV%)** | | | | | | |
| V2 | 117.5 | 12.6 |  | 117 | 89.5 | 165 |
| **Residual variability** | | | | | | |
| Proportional (%) | 35.9 | 3.6 |  | 35.6 | 33.7 | 37.6 |
| Additive (ng/mL) | 1.463 | 9.7 |  | 1.45 | 1.23 | 1.69 |

CL, clearance; V2, central volume of distribution; V3, peripheral volume of distribution; Ka, absorption rate constant; Q, intercompartment clearance; F1, relative bioavailability; ALAG, absorption lag time; DOSE-Ka: Effect of dose amount (mg) to Ka; FOOD-Ka: Effect of food to Ka when fed status compared with fasted status; FOOD-F1: Effect of food to F1 when fed status compared with fasted status; Ritonavir-F1: Effect of relative administration time of ritonavir on F1 (Administration time of ritonavir was before simnotrelvir, compared with simultaneous administration).

^a^ CL (L/h) = 22.6 × (CRCL/113)^0.39^

^b^ Ka (h^-1^) = 0.755 × DOSE^-0.229^ ×“FOOD-Ka”

^c^ F1=1 × “Ritonavir-F1” × “FOOD-F1”

Table S6. Dose simulation result of simnotrelvir/ritonavir in fasted status after Model2.

|  | **750/100 mg BID, first dose** | **750/100 mg BID, steady state** |
| --- | --- | --- |
| C_trough_ (ng/mL), median (10^th^-90^th^) | 924 (424-1780) | 1249 (556-2497) |
| AUC_0-12_ (ng*h/mL), median (10^th^-90^th^) | 26362 (17028-40123) | 37758 (25239-56826) |
| %Subjects C_trough_>EC_90_^a^ | 96.5 | 97.9 |

Note: ^a^ The EC_90_ for the simnotrelvir against the Omicron virus was 274 ng/mL.

Table S7. Description of PK exposure parameters of subjects in Phase II/III with estimation of Model 2 included in the ER study.

|  | **AUC(ng*h/mL)**  **(predicted)** | **C_max_(ng/mL)**  **(predicted)** | **C_trough_(ng/mL)**  **(observed)** |
| --- | --- | --- | --- |
| N | 95 | 95 | 95 |
| Mean±SD | 45187.4±9708.1 | 6382.6±1680.0 | 1603.6±775.4 |
| Median (minimum- maximum) | 45309.7 (25177.9-78472.9) | 5965.5 (3153.4-13103) | 1522.3 (44.9-4639.7) |

N, number of subjects; SD, standard deviation;

**References**

1. Yang XM, Yang Y, Yao BF, Ye PP, Xu Y, Peng SP, et al. A first-in-human phase 1 study of simnotrelvir, a 3CL-like protease inhibitor for treatment of COVID-19, in healthy adult subjects. Eur J Pharm Sci. 2023 Dec 1;191:106598.

2. Wang F, Xiao W, Tang Y, Cao M, Shu D, Asakawa T, et al. Efficacy and safety of SIM0417 (SSD8432) plus ritonavir for COVID-19 treatment: a randomised, double-blind, placebo-controlled, phase 1b trial. Lancet Reg Health West Pac. 2023 Sep;38:100835.

3. Cao B, Wang Y, Lu H, Huang C, Yang Y, Shang L, et al. Oral Simnotrelvir for Adult Patients with Mild-to-Moderate Covid-19. N Engl J Med. 2024 Jan 18;390(3):230-41.

4. Hooker AC, Staatz CE, Karlsson MO. Conditional weighted residuals (CWRES): a model diagnostic for the FOCE method. Pharm Res. 2007 Dec;24(12):2187-97.

5. Lindbom L, Ribbing J, Jonsson EN. Perl-speaks-NONMEM (PsN)--a Perl module for NONMEM related programming. Comput Methods Programs Biomed. 2004 Aug;75(2):85-94.

6. Comets E, Brendel K, Mentré F. Computing normalised prediction distribution errors to evaluate nonlinear mixed-effect models: the npde add-on package for R. Comput Methods Programs Biomed. 2008 May;90(2):154-66.

7. Ding J, Wang Y, Lin W, Wang C, Zhao L, Li X, et al. A population pharmacokinetic model of valproic acid in pediatric patients with epilepsy: a non-linear pharmacokinetic model based on protein-binding saturation. Clin Pharmacokinet. 2015 Mar;54(3):305-17.

8. van der Meer AF, Marcus MA, Touw DJ, Proost JH, Neef C. Optimal sampling strategy development methodology using maximum a posteriori Bayesian estimation. Ther Drug Monit. 2011 Apr;33(2):133-46.
